# Microgel Aspect Ratio Influences Injectable Granular Hydrogel Scaffold Pore Structure and Cellular Invasion for Tissue Repair

**DOI:** 10.1002/advs.202511513

**Published:** 2025-09-12

**Authors:** Gabriel J. Rodriguez‐Rivera, Siddharth Sharma, Chima V. Maduka, Sara Boyd, Amy R. Perry, Nikolas Di Caprio, Lindsay Riley, Connor E. Miksch, Daeyeon Lee, Tatiana Segura, David Issadore, Jason A. Burdick

**Affiliations:** ^1^ BioFrontiers Institute University of Colorado Boulder Boulder CO 80309 USA; ^2^ Department of Chemical and Biomolecular Engineering University of Pennsylvania Philadelphia PA 19104 USA; ^3^ Materials Science & Engineering Program University of Colorado Boulder Boulder CO 80309 USA; ^4^ Department of Bioengineering University of Pennsylvania Philadelphia PA 19104 USA; ^5^ Department of Biomedical Engineering Duke University Durham NC 27708 USA; ^6^ Department of Chemical and Biological Engineering University of Colorado Boulder Boulder CO 80309 USA; ^7^ Present address: Department of Chemical and Biochemical Engineering Villanova University Villanova PA 19085 USA

**Keywords:** cellular invasion, granular hydrogels, porosity, tissue repair, void fraction

## Abstract

Granular hydrogels are emerging as an important class of scaffolds for biomedical applications, due to their injectability and pore structure to support cellular infiltration. Past research has primarily focused on spherical microgels, which allows limited control over granular hydrogel pore size and void volume fraction; however, investigation into microgels with higher aspect ratios has allowed even higher porosity. This study explores the impact of hyaluronic acid microgel aspect ratio (ranging from 3 to 5) on granular hydrogel porosity and cellular interactions. Both simulations and experimental results show increased void volume fractions and pore sizes in granular hydrogels formed from rod‐like microgels when compared to volume‐matched spherical microgels, which results in increased cellular invasion with an endothelial cell spheroid migration assay. Injection of the hydrogels into a confined space alters particle packing and void space, but porosity is still higher when rod‐like microgels are used, which results in increased cellular invasion when injected subcutaneously. Finally, the highest aspect ratio microgels are used as injectable granular hydrogels to treat myocardial infarction in rats and show reduced infarct area and enhanced functional outcomes when compared to untreated controls. This work provides further insight into microgel shape considerations for engineered granular hydrogels.

## Graphical abstract

Granular hydrogels are being developed for the repair of tissues, mostly with the use of spherical microgels. Here, injectable granular hydrogels formed from anisotropic particles (i.e., rod‐like) are explored to improve cellular invasion by increasing scaffold void volume fraction. One formulation is then investigated for the repair of cardiac tissue after myocardial infarction.

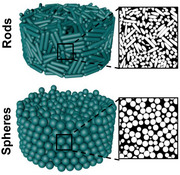

## 1 Introduction

Granular hydrogels are formed through the assembly of hydrogel microparticles (i.e., microgels) and are now being used widely in tissue engineering due to their modularity, injectability, and unique ability to control void volume fraction and pore size, which are critical factors for cellular infiltration and tissue vascularization.^[^ ^1^, ^2^ ^]^ Early studies used spherical microgels to build granular scaffolds suitable for exogenous tissue growth,^[^ ^3^ ^]^ which then advanced to the development of injectable microporous annealed particle (MAP) scaffolds for endogenous wound healing.^[^ ^4^ ^]^ In MAP scaffolds, jammed microgels are delivered through a needle, and when annealed, they interlink to form a porous scaffold at the wound site. The last decade has seen rapid growth in the development and use of granular hydrogels, particularly due to their intriguing properties, such as shear‐thinning and shear‐recovery behavior^[^ ^5^, ^6^ ^]^ (i.e., viscous flow under applied stress and recovery when stress is released^[^ ^7^ ^]^), which has been leveraged in the development of tissue engineering scaffolds,^[^ ^2^, ^8^ ^]^ cell and drug encapsulation vehicles,^[^ ^9^ ^]^ and inks for 3‐dimensional (3D) printing applications.^[^ ^2^, ^10^ ^]^ Most previous studies have focused on spherical microgels, which limits the modularity of granular hydrogels and alterations in structural features such as void volume fraction, which is commonly referred to as porosity.

To enhance endogenous tissue repair with scaffolds, where cells invade from local tissues to support tissue formation, features such as void volume fraction and pore size are particularly important,^[^ ^11^ ^]^ as they guide important cell–cell and cell–material interactions, as well as tissue remodeling.^[^ ^12^, ^13^ ^]^ Traditional hydrogels have pore sizes in the nanometer range, which restricts the infiltration of cells (≈10 and 20 µm) without degradation.^[^ ^14^ ^]^ For neovascularization, studies with porous hydrogel scaffolds found that pore sizes of ≈50–150 µm allow mature vascularized tissue growth, while pores of ≈25–50 µm limit the vascularization to the outer portions of scaffolds.^[^ ^15^ ^]^ With regards to granular hydrogels, a recent review reported that granular hydrogels comprised of spherical microgels have mean pore sizes of ≈20–50 µm,^[^ ^16^ ^]^ which has spurred interest in methods to further enlarge pore size. One option to increase pore size is to increase the size of microgels used, which has been shown both theoretically and experimentally.^[^ ^11^, ^13^, ^17^ ^]^ However, increasing the microgel diameter does not change the scaffold void fraction and may result in challenges in material injection through smaller diameter needles.

Void volume fraction, which is the open void space available within the scaffold for cells to migrate, proliferate, and remodel is as critical as pore size. The importance of void volume fraction on tissue engineering scaffolds and different fabrication techniques has been reviewed in several articles previously.^[^ ^16^, ^18^, ^19^, ^20^ ^]^ For example, one study found that a human elastin scaffold with an average void volume fraction of 14.5% and a mean pore size of 8 µm only promoted cell proliferation across the scaffold surface, while increasing the void volume fraction to 34.4% and mean pore size to 11 µm enabled infiltration of dermal fibroblasts throughout the entire scaffold.^[^ ^21^ ^]^ Another study using highly porous scaffolds showed that scaffolds with large pore sizes (200 to 250 µm) and void volume fraction (86%) enabled fibroblast proliferation, although proliferation was improved for scaffolds with smaller pore sizes (100 to 150 µm) by increasing void volume fraction to 91%.^[^ ^22^ ^]^ Previous techniques to control scaffold void volume fraction, such as 3D printing,^[^ ^23^, ^24^ ^]^ electrospun fiber scaffolds,^[^ ^25^, ^26^, ^27^ ^]^ and inverse opal scaffolds,^[^ ^28^ ^]^ are unsuitable as injectable scaffolds, which motivates the use of granular hydrogels. The void volume fraction of granular hydrogel scaffolds comprised of spherical microgels is often in the mid to lower range (<50%)^[^ ^16^ ^]^ and is theoretically ≈36% for randomly packed spheres;^[^ ^29^ ^]^ thus, further increasing the void volume fraction of granular scaffolds has become a critical goal.

There has been significant interest in understanding how pore features of granular hydrogels influence cell invasion and behavior.^[^ ^7^, ^13^, ^30^, ^31^, ^32^, ^33^, ^34^ ^]^ While there are a number of parameters that can be altered to change void fraction (e.g., extent of jamming), multiple groups including our own have explored the fabrication of granular hydrogels using non‐spherical microgels,^[^ ^16^ ^]^ including with rod‐like anisotropic microgels due to their unique geometry (increased aspect ratio (AR)).^[^ ^6^, ^35^, ^36^ ^]^ Riley et al. showed that ellipsoids pack more tightly than spheres in random packing configurations, while rods produce larger void volume fractions.^[^ ^37^, ^38^, ^39^ ^]^ Previous work from our lab showed that granular hydrogels from rod‐like particles (AR 2.2) have void volume fractions comparable to spherical microgels of similar volume, but still support increased cell invasion, likely due to anisotropic pore shapes that can guide cellular infiltration.^[^ ^5^ ^]^ In vitro studies with elongated microrods having complementary chemistries that react to form scaffolds with large porosities have also shown promise for 3D cell culture;^[^ ^35^, ^40^ ^]^ however, this approach relies on fast kinetics of microgel annealing, resulting in fluctuating porosities (40% to 70%) with no clear differences when AR is changed. Contrary to previous studies and theoretical work, studies of even higher AR (up to 20) did not find differences in void fraction in granular hydrogels when compared to those fabricated with rod‐like microgels with AR lower than 10.^[^ ^40^, ^41^ ^]^ However, these studies only investigated the packing of microgels by centrifugation into open containers. Thus, additional work is needed to advance our understanding of microgel design on the development of injectable granular hydrogels that support cellular invasion in the context of tissue repair in vivo.

Percolation studies, molecule diffusion, and analysis of 2D images have been helpful tools to assess granular hydrogel void fraction, and newer computational tools now enable a thorough assessment of 3D pore features.^[^ ^17^, ^38^ ^]^ Prior simulations showed that the AR of rod‐like particles affects their packing, with shorter particles of AR 1.5 packed tightly (≈30% void volume fraction) and void volume fraction increasing with increased AR.^[^ ^42^, ^43^ ^]^ Despite this knowledge, experimental studies have reported conflicting results in terms of the impact of AR on the porosity of granular hydrogels from rod‐like particles.^[^ ^35^, ^40^, ^41^ ^]^ This is likely due to differences in the granular hydrogel compositions, including the methods used to jam microgels into granular hydrogels, microgel properties such as mechanics that can alter microgel jamming (e.g., rigid rods vs those than can deform when centrifuged), and differences in whether the microgels are annealed. Further, investigating the granular hydrogel structure after injection has been largely neglected in the prior work on microgel AR, despite the potential impact of injection into a constrained volume on granular material properties. Specifically, the void volume fraction is typically assessed only after packing of microgels by centrifugation or vacuum;^[^ ^5^, ^44^ ^]^ however, the injection process (flow through the confined space in a needle) and then accumulation at the injection site will likely alter packing.

We hypothesized that increasing the AR of microgels over our previous study of AR 2.2 would allow control over both void volume fraction and pore sizes within granular hydrogels and result in granular hydrogels where the void volume fraction is greater with rod‐like microgels over their spherical counterparts. To investigate this, we used microfluidics to synthesize microgels with ARs that vary from 3 to 5 and used combined experimental and computational approaches to investigate void volume fractions and pore features and their influence on cell invasion within granular hydrogels. To address previous concerns related to injectability, we then characterized the granular hydrogels after injection, first for structure and then for cellular invasion after delivery to the subcutaneous space. Finally, the formulation with the highest AR was investigated in a more complex model of myocardial infarction (MI), which we believe is the first time that a granular hydrogel with high AR microgels has been used for endogenous tissue repair after MI. Overall, this work addresses the crucial need to understand and engineer injectable granular hydrogel scaffolds with controlled void volume fractions and pore sizes for tissue repair.

## 2 Results

### 2.1 Fabrication of Microgels and Granular Hydrogel Scaffolds

Microgels can be fabricated through various methods, such as extrusion fragmentation, batch emulsion, microfluidics, and in‐mold fabrication.^[^ ^5^, ^45^, ^46^, ^47^, ^48^ ^]^ Microfluidics, a continuous fabrication process with excellent particle size control, was selected to fabricate microgels with both rod‐like and spherical shapes, while minimizing particle polydispersity. Specifically, microgels were fabricated using norbornene‐modified hyaluronic acid (NorHA). HA was selected as it is a naturally derived polymer that interacts with cells via CD44 receptors, and it has been previously shown to promote angiogenesis,^[^ ^49^, ^50^ ^]^ reduce fibrotic responses, and improve cardiac function without potent angiogenic factors.^[^ ^51^ ^]^

**Figure 1:**
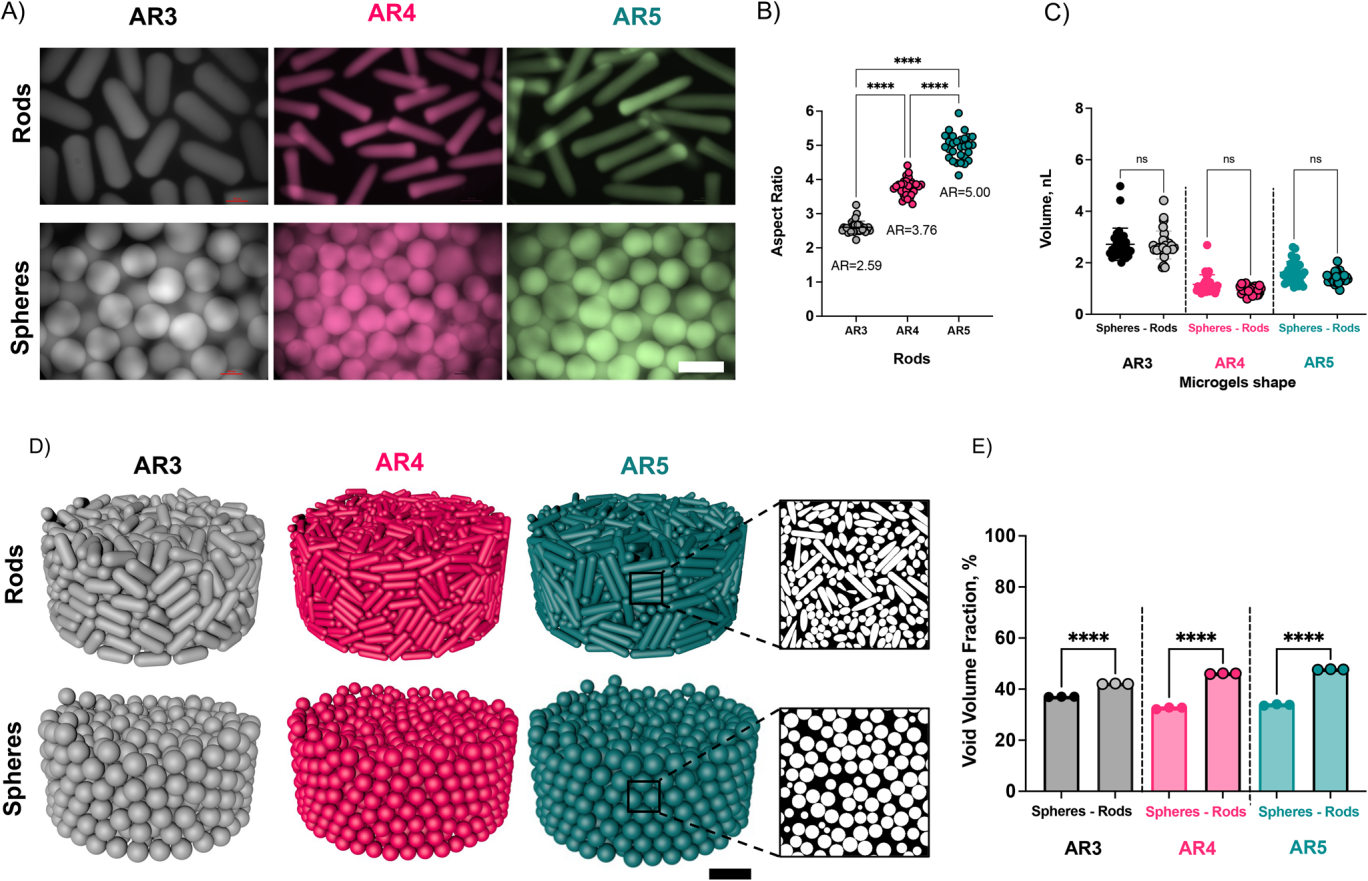
Microgel synthesis, characterization, and simulation of packing into granular hydrogel scaffolds. A) Representative images of rod‐like (with different aspect ratios (ARs): AR3, AR4, AR5) and spherical microgels of equivalent volumes (also labelled as AR3, AR4, and AR5 for clarity in comparison to their rod‐like counterpart, although spherical microgels exhibit an AR1). Scale bar = 200 µm. Quantified B) ARs and C) volumes of both rod‐like and spherical microgels (*n* = 30). Note that microgels of various ARs exhibited different volumes, so direct comparisons across the various ARs are limited, and statistical analyses are focused on comparing rod‐like microgels with their volume‐matched spherical counterpart. D) Representative images and E) quantification of void volume fraction (*n *= 3) from Cinema 4D dynamic simulations of granular hydrogels from rod‐like and spherical microgels, using experimental inputs on specific microgel dimensions. Scale bar = 300 µm; Mean (SD); ns: not significant, *p* > 0.05; ****p < 0.0001.

Using single‐channel microfluidic devices, droplets of an aqueous precursor solution containing the NorHA macromer, a dithiol crosslinker, and a photoinitiator were generated within a continuous oil phase. For rod‐like microgels, the droplet was confined into a narrow channel and crosslinked on‐chip with light to stabilize the structure before washing from oil, whereas spherical microgels were formed without confinement. Rod‐like microgels were fabricated with aspect ratios (ARs) of ≈3, 4, and 5, denoted AR3, AR4, and AR5 (**Figure** **1**A,B). The mean diameter and standard deviation of rod‐like microgels were between ≈68 ± 5 and 107 ± 8 µm, and the equivalent diameter of spherical microgels was between ≈129 ± 12 and 172 ± 12 µm (actual dimensions shown in Figures S1 and S2, Supporting Information). Fluorescein isothiocyanate (FITC) dextran was incorporated into the microgels for fluorescence microscopy visualization, and the microgels were designed for similar volumes between rod‐like and spherical microgels to enable investigation of the influence of microgel shape on granular hydrogel properties (Figure 1C).

To generate rod‐like microgels with varying AR using the same channel geometry, it was necessary to vary the droplet volumes used within the microfluidic device. Specifically, rod‐like microgels of different ARs were generated by varying the flow rate ratio of the dispersed and continuous phases, which varied the droplet size. With this, the droplets used to fabricate the longer AR rod‐like microgels (AR4 and AR5) experienced different shear forces due to operating in a lower capillary number, which resulted in a sharper leading edge, flattened trailing edge, and a shorter overall mean cross‐sectional diameter.^[^ ^52^ ^]^ After the fabrication of microgel batches that met the criteria for the desired ARs, spherical microgels were then fabricated in microfluidic devices (without confinement in the narrow channel) with the identical macromer formulation, with appropriate droplet volumes controlled through microfluidic parameters to match the final volumes of each AR (Figure 1C).

To better understand the influence of microgel AR on granular hydrogel void volume fraction, rigid body dynamic simulations in Cinema4D were performed using the experimental rod‐like and spherical microgel dimensions, with packing simulated into a cylindrical boundary via gravity (Figure 1D). Although there are some limitations to this approach considering that the microgels may not behave as rigid bodies and the packing may be driven through other forces beyond gravity, this provides insight into how microgels pack into granular structures with varied ARs. The simulations demonstrated increased void volume fraction with rod‐like microgels when compared to spherical microgels across the various ARs, with rod‐like microgels consistently exhibiting higher void volume fraction than volume equivalent spherical microgels (Figure 1E). The mean void volume fraction for granular hydrogels from spherical microgels varied between 32.6 ± 0.4 to 37 ± 0.1%, which is within the range of previously reported values for random packing of spheres.^[^ ^29^ ^]^ In comparison, the granular hydrogels from rod‐like microgels ranged between 42.1 ± 0.1 to 47.7 ± 0.1%. In general, the simulations confirmed predictions by others that there are increased void volume fractions for assemblies from anisotropic particles when compared to spherical particles.

### 2.2 Microgel Scaffold Void Space and Pore Size Increase as AR Increases

**Figure 2:**
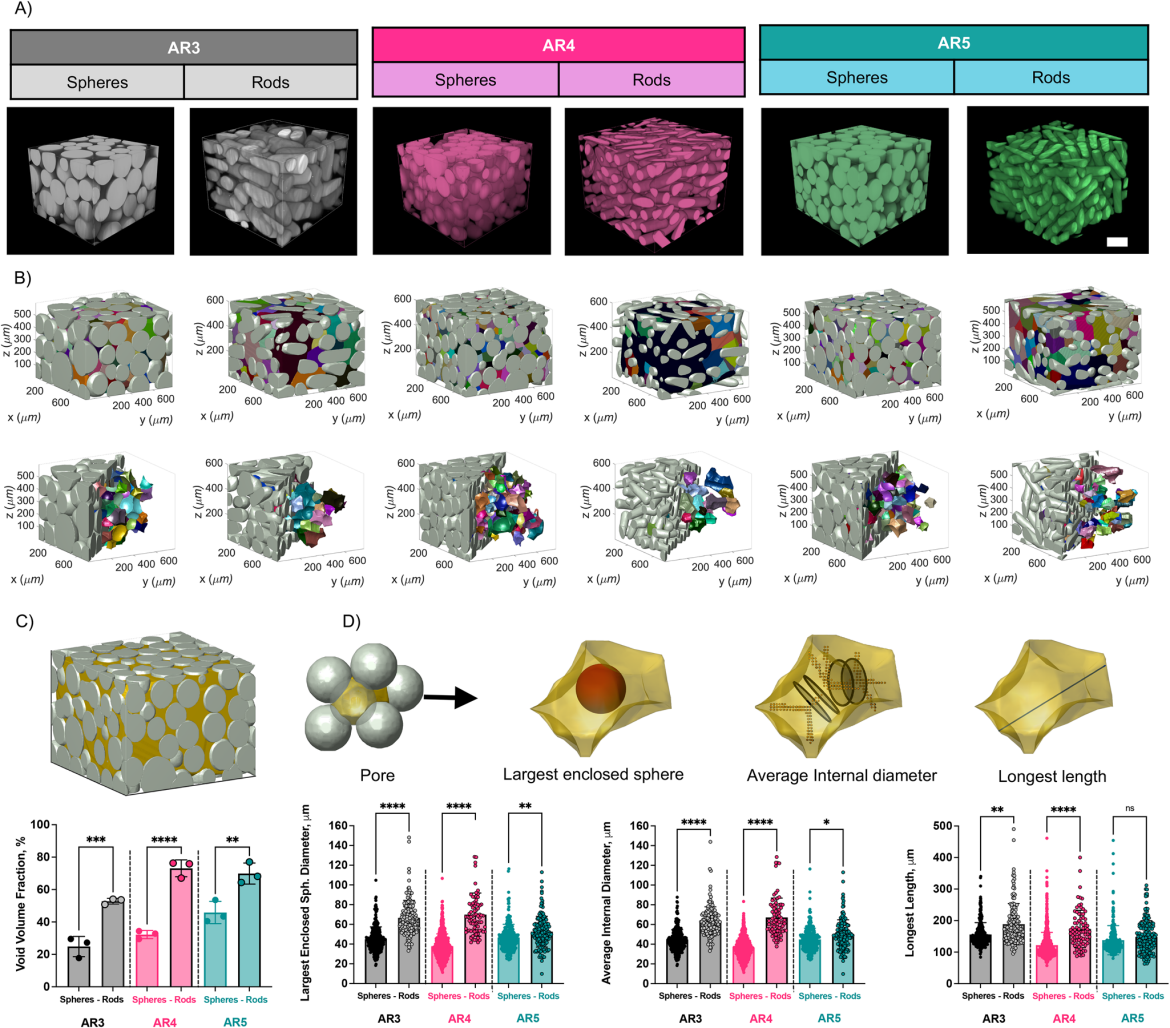
Impact of microgel shape and aspect ratio on granular hydrogel scaffold void volume fraction and pore size. A) Representative confocal images of granular hydrogel scaffolds fabricated from rod‐like and spherical microgels (778 × 778 × 600 micron z‐stacks, 300 images per stack). B) Representative images of pore segmentation generated by Local Void Analysis using Medial Axis by Particle configuration (LOVAMAP) code across the formulations, where only interior pores are colored as individual pores and considered for analysis. C) Descriptor (top) and quantified void volume fraction (bottom) of granular hydrogels via measurements of void space (*n* = 3 independent scaffolds, average of 2 locations/stacks per scaffold). D) Descriptors (top) and quantification (bottom) of individual pores segmented by LOVAMAP, which includes the largest enclosed spheres, average internal diameter, and longest length. The number of internal pores identified by LOVAMAP varied across samples (3 independent scaffolds, 2 locations/scaffold, Spheres AR3 *n *= 335, Rods AR3 *n* = 152, Spheres AR4 *n* = 949, Rods AR4 *n *= 80, Spheres AR5 *n* = 281, Rods AR5 *n* = 166). Mean (SD); ns: not significant, *p* > 0.05; **p < 0.01; ***p < 0.001; ****p < 0.0001.

Microgels were jammed into granular hydrogels using centrifugation and imaged via confocal microscopy (**Figure** **2**A). The resultant z‐stack data were then analyzed via the Local Void Analysis using Medial Axis by Particle configuration (LOVAMAP) software, with details that have been previously published.^[^ ^17^ ^]^ LOVAMAP identified the void fraction in three dimensions, segmented it into individual 3D pores, and provided multiple descriptors to elucidate 3D pore dimensions (Figure 2B–D).^[^ ^53^ ^]^ The impact of AR on void volume fraction was then calculated and found to be higher for all granular hydrogels composed of rod‐like microgels compared to those made from spherical microgels of similar volume (Figure 2C). This observation corroborates our simulations (Figure 1D), as well as previous simulations of random rod packing.^[^ ^42^, ^43^ ^]^ The mean void volume fractions of granular hydrogels from spherical microgels were between 24.8 ± 6.2 to 45.8 ± 6.9%, whereas the mean void volume fractions of granular hydrogels from rod‐like microgels were 52.8 ± 1.8 to 69.8 ± 6.5%. A two‐way ANOVA analysis was used to compare the impact of both the Shape factor (Spheres vs Rods) and the AR Factor (AR 3 – 5) on the void volume fraction, which showed that both factors and their interaction are statistically significant (Table S1, Supporting Information). Contrary to earlier reports that showed no difference in void volume fraction between granular hydrogels from rod‐like and spherical microgels at a low AR of 2.2,^[^ ^6^ ^]^ these findings indicate that with higher ARs the void volume fraction increases.

LOVAMAP outputs multiple descriptors of 3D pores that can be used to assess and compare the 3D pore characteristics for granular hydrogel scaffolds. LOVAMAP software can segment simulated^[^ ^17^ ^]^ and real scaffolds from microscope z‐stack images of the scaffold.^[^ ^54^ ^]^ Figure 2B illustrates representative images of the granular scaffolds after pore segmentation. Because microscope images segment particles and the void arbitrarily, the 3D pores at the edge of the image may not represent the full 3D pore; thus, external 3D pores were excluded from analyses and only internal 3D pores were analyzed. Two 3D pore descriptors that provided insight into 3D pore size are the largest enclosed sphere diameter and the average internal diameter (Figure 2D). Both descriptors were larger for granular hydrogels from rod‐like microgels compared to those comprised of spherical microgels of equal volume. Moreover, the average internal pore diameters were between 51 and 67 µm for rod‐like microgels (**Table** **1**), which is above the 50 µm range for enhanced cell and tissue infiltration described in the literature.^[^ ^16^ ^]^ In contrast, the pore diameters were much smaller for spherical particles with median values between 36 and 45 µm (Table 1). However, other studies comparing spherical hydrogel scaffolds found enhanced vascularization in vivo with smaller pore sizes of equivalent median diameter of ≈20 µm.^[^ ^11^ ^]^ Here, we will compare the spherical and rod‐like microgels of similar volume to assess if different microgel shapes influence cell infiltration.

**Table 1:** Mean void volume fraction, median, and interquartile range for granular scaffolds pore descriptors.

Descriptors	AR3		AR4		AR5	
	Spheres	Rods	Spheres	Rods	Spheres	Rods
Scaffold void volume fraction (%)	24.8	52.8	32.3	73.1	45.8	69.8
Number of internal pores LOVAMAP segmented	335	152	949	80	281	166
Largest enclosed sphere diameter (µm)	43.5 (36.7–50.8)	64.4 (55.18–75.9)	36.4 (29.1–44.9)	67.3 (53.5–80.2)	44.7 (37.3–52.5	50.8 (43.2–60.4)
Average internal diameter (µm)	42.7 (36.7–49.5)	61.5 (54.4–70.4)	35.8 (28.8–42)	62.4 (53.3–77.7)	43.5 (36.8–50.3)	49.0 (40.7–58)
Longest pore length (µm)	146 (132–168)	172 (142–214)	110 (97–134)	164 (135–206)	126 (112–149)	139 (111–180)
Aspect ratio	3.54 (3.05–4.10)	2.87 (2.51–3.27)	3.33 (2.89–3.92)	2.62 (2.04–3.09)	2.99 (2.66–3.46)	2.74 (2.36–3.47)

In terms of pore elongation, the longest 3D pore end‐to‐end length (Figure 2D) was higher for 3D pores generated from rod‐like microgels with AR3 and AR4, but not statistically different than the AR5 microgels when compared to spherical microgels. Nevertheless, all granular scaffolds from rod‐like microgels had higher median and interquartile 3D pore average internal diameters and end‐to‐end lengths than their equivalent scaffolds from spherical microgels. The median 3D pore end‐to‐end length was within 110 and 146 µm for granular hydrogels from spherical microgels and 139 to 172 µm for those from rod‐like microgels. Although the length was higher for all rod‐like microgels, interestingly, it was not statistically significant for AR5. Counterintuitively, the ARs of 3D pores (end‐to‐end length/average internal diameter) within granular scaffolds were lower for scaffolds formed from rod‐like microgels than for spherical microgels. For reference, an AR for a 3D pore = 1 would mean a spherical pore. This finding in AR for 3D pores could be due to the pore internal diameters increasing much more than their length when switching from spherical to rod‐like microgels or local alignment of the rods. In fact, this was observed from the segmentation, with broader pores from rod‐like microgels that resulted in larger diameter pores. Additional pore descriptors (pore volume, surface area, and pore isotropy) are shown in Figure S3 (Supporting Information).

### 2.3 Cell Sprouting into Granular Hydrogel Scaffolds Increases with Higher AR Microgels

**Figure 3:**
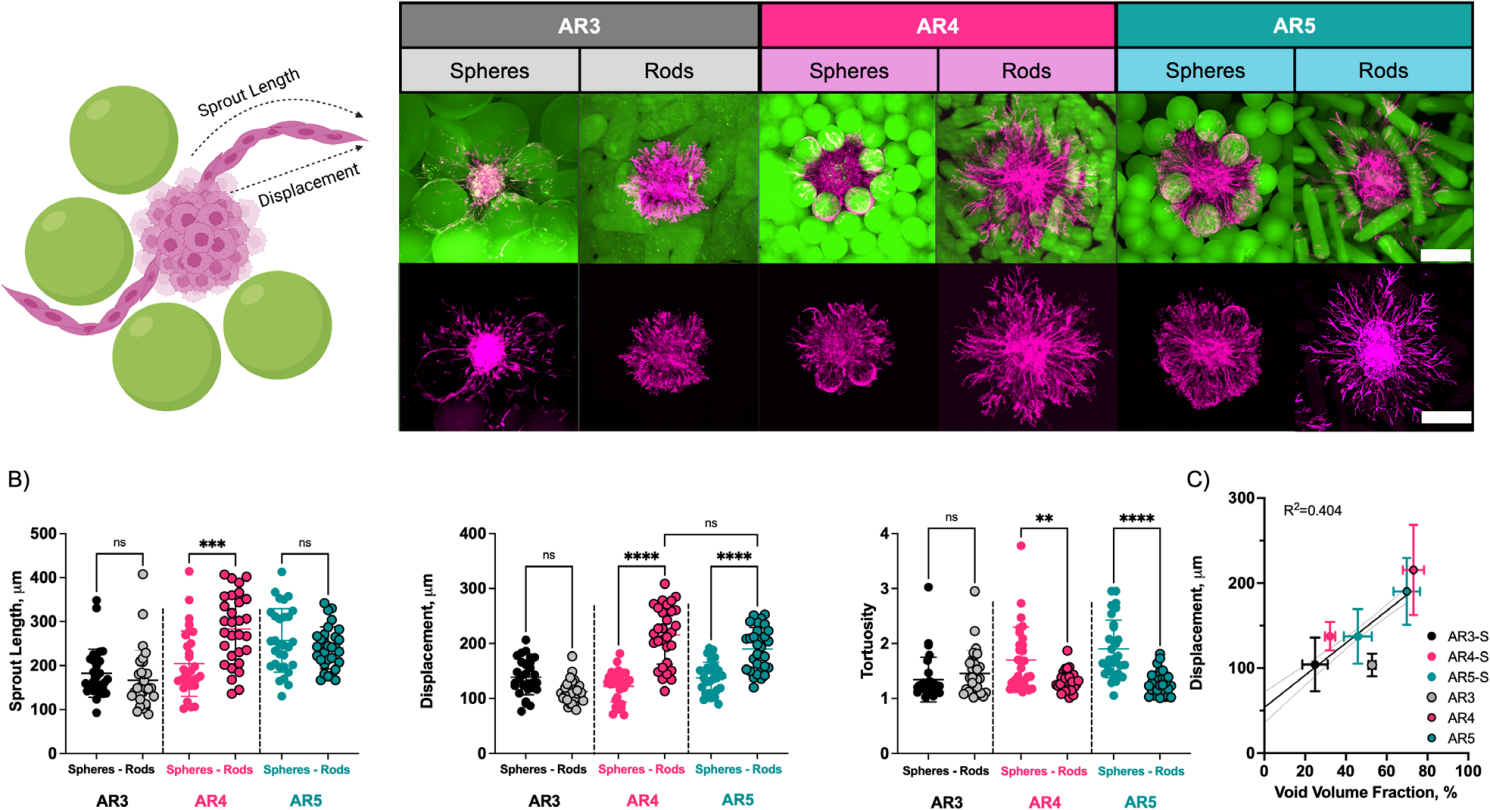
Influence of microgel shape and aspect ratio on cell sprouting into granular hydrogels in vitro. A) Schematic and representative projection of cell sprouting (confocal images, Alexa Fluor 647 Phalloidin, FITC‐Dextran, z‐stacks) within granular hydrogels fabricated from rod‐like and spherical microgels at various ARs. Scale bars = 200 µm. B) Quantification (Manual Tracking tool from ImageJ (*n* = 30, 3 replicates, 10 sprouts per replicate)) of cell sprouting metrics (sprout length, displacement, tortuosity) from spheroids of endothelial and mesenchymal stromal cells into granular hydrogels. C) Correlation between sprout displacement into granular scaffolds and void volume fraction. Mean (SD); ns: not significant, *p* > 0.05; **p < 0.01; ***p < 0.001; ****p < 0.0001.

One of the main advantages of granular hydrogel scaffolds is intrinsic porosity, or void space between particles, which ideally allows and guides cell invasion into scaffolds, such as via angiogenesis.^[^ ^55^, ^56^ ^]^ An in vitro assay was developed to assess the impact of microgel shape and AR on cell sprouting,^[^ ^5^ ^]^ where spheroids comprising human umbilical vein endothelial cells and human mesenchymal stromal cells are embedded and cultured within granular hydrogel scaffolds. All granular hydrogels were packed using similar parameters, and spheroids were formed from the same cell culture. After 3 days, cell sprouting was analyzed using confocal microscopy and Fiji software (**Figure** **3**A).

Granular hydrogels of high AR rod‐like microgels showed increased sprout displacement (end‐to‐end distance) and decreased tortuosity compared to granular hydrogels of equivalent volume spherical microgels (Figure 3B). Displacement is an indication of how far into the scaffold the sprouts go from the initial spheroid. For AR4 and AR5 rod‐like microgels, this distance was within 215 ± 53 to 190 ± 39 µm, which is greater than values of 122 ± 28 and 137 ± 28 µm measured for spherical microgels. The mean sprout length for the higher AR rod‐like microgels (AR5) was comparable to spherical microgels (Rods 240 ± 48 vs Spheres 257 ± 73 µm), but the tortuosity was much lower for the rod‐like microgels, indicating that the lower tortuous path between pores allows the sprouts to displace further into the granular scaffolds. There were no significant differences between the shorter rod‐like microgels (AR3) and equivalent spheres. Interestingly, there was good correlation between sprout displacement and scaffold void volume fraction (*R* ^2 ^= 0.404), highlighting the significant contribution of void volume fraction for cell invasion into granular scaffolds with pore sizes within ≈40–60 microns in diameter (Figure 3C).

Within our study, microgel AR and volume changed across the different comparisons from AR3 up to AR5. Both factors (microgel AR and volume) could contribute to the overall changes in pore space and, therefore, cell migration. In this case, we observed an enhancement in AR4 and AR5 granular hydrogels when compared to their equivalent spheres. Rod‐like microgels may also improve cell infiltration into scaffolds by providing a path for cell migration along the longer axis. This is coupled with pore geometries and interconnectivity to guide cellular infiltration. Notably, we observed a decrease in tortuosity for sprouts within granular hydrogels from rod‐like microgels of higher aspect ratio, indicating a more linear, less tortuous path.

### 2.4 Granular Hydrogel Flow and Injectability

**Figure 4:**
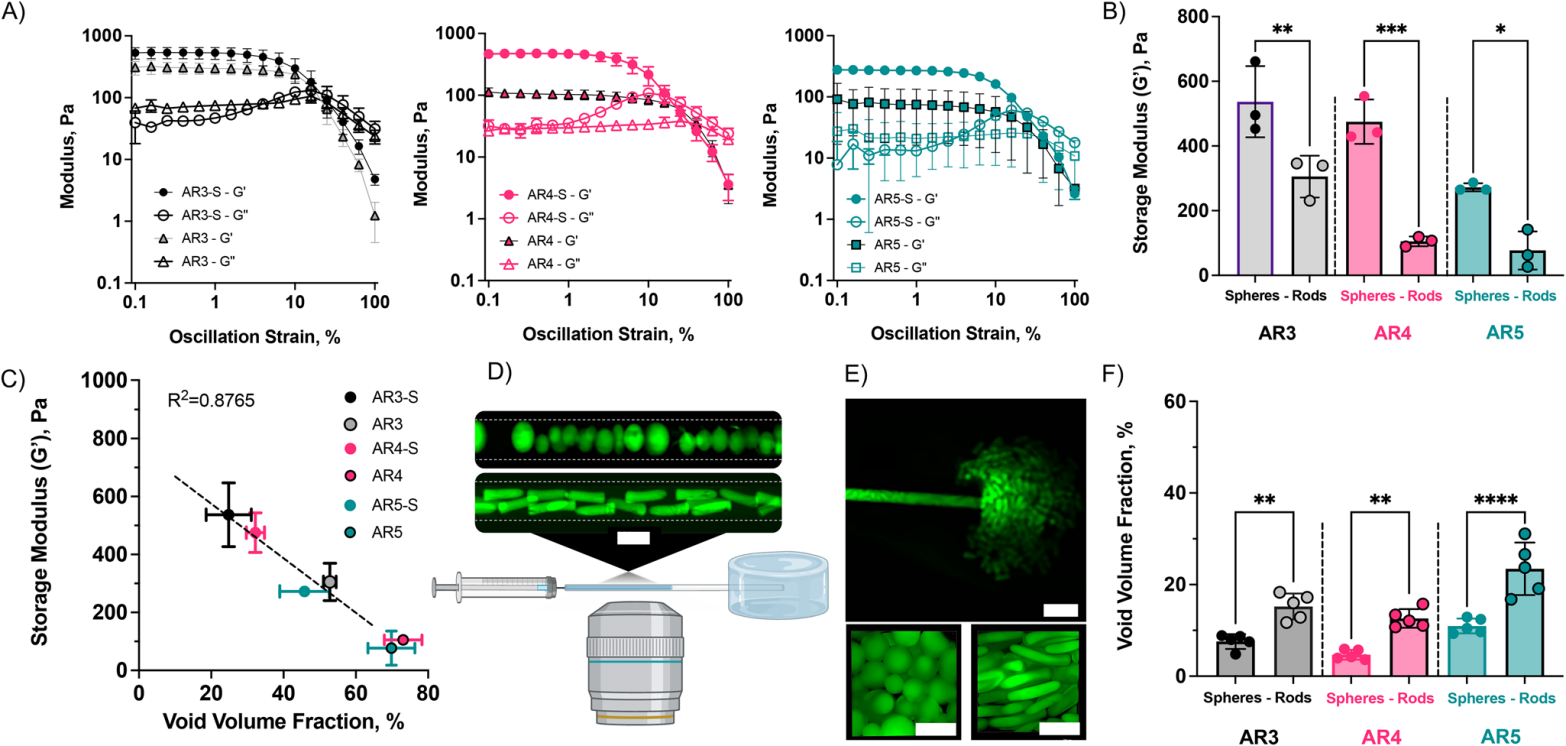
Granular hydrogel rheological properties based on microgel shape and aspect ratio. A) Rheological profiles (strain sweep, 1 Hz) and B) quantified storage modulus (G′) of granular hydrogels with varied AR and shape (*n* = 3). C) Correlation between storage modulus and scaffold void volume fraction across groups (*n *= 3). Confocal images of D) spherical and rod‐like (AR5) microgels flowing through a capillary tube, Scale bar = 250 µm, and E) after injection into gelatin hydrogels, Scale bars = 500 µm. F) Quantification of void volume fraction of microgels after injection (*n* = 5). Mean (SD); ns: not significant, *p* > 0.05; *p < 0.05; **p < 0.01; ***p < 0.001; ****p < 0.0001.

An injectable scaffold must be deliverable through a small catheter or syringe for tissue engineering applications, which typically range in the order of 10^2^ to 10^3^ µm in diameter. A potential challenge to granular systems of high AR microgels is their impact on injectability. **Figure** **4**A shows shear‐yielding behavior across the different granular gels. The yielding behavior is indicative that at higher strains, the hydrogels have more fluid‐like properties, suitable for injection. Granular hydrogels from rod‐like microgels exhibited lower storage moduli (≈100–300 Pa) than for spherical microgels (≈200–500 Pa), especially for higher AR (Figure 4B). This is the opposite trend observed in a previous study with shorter rod‐like microgels,^[^ ^6^ ^]^ and we hypothesized that this is likely due to the larger void fraction that allows easier microgel flow through interstitial spaces (Figure 4C) and the higher water content. This highlights the relevance of AR as a design criterion for these injectable scaffolds.

Using a strain sensor, we determined the force required to inject granular hydrogels through a 27G needle (ID ≈ 230 µm, Figure S4, Supporting Information). The force was less than 2 N, indicating that minimal effort is required for manual injectability.^[^ ^57^ ^]^ This is well within the <50 N acceptable limit of ISO 7886‐1 (Sterile hypodermic syringes for single use).^[^ ^58^ ^]^ High AR rod‐like microgels required less force to inject than spheres, possibly due to their ability to align parallel to the flow direction in the channel and enhanced void volume fraction. To better understand microgel flow through a channel with a diameter close to the size of a needle, we used confocal microscopy to track microgel flow in a 2‐microliter capillary tube with a 280 µm internal diameter (Figure 4D). This is slightly larger than the inner diameter for a 27G needle. Confocal microscopy showed elongated rod‐like microgels aligning with the channel flow, while variability in spherical microgel diameter increased flow resistance, likely due to the presence of larger diameter spheres (Figure 4D).

During injection, microgels must flow through the needle and then accumulate within a constrained volume (as a tissue), which may alter microgel jamming and is in contrast to the more open space that is often used for in vitro analysis. To investigate this, we fabricated gelatin hydrogels on a glass slide, injected the microgels into the gelatin via the same capillary tube, and analyzed z‐stacks to determine void volume fraction using confocal microscopy (Figure 4E). The void volume fraction of granular hydrogels from spherical microgels was between 4.69 ± 1.06 and 11 ± 1.6%, whereas the void volume fraction of granular hydrogels from rod‐like microgels was between 12.6 ± 2 to 23.5 ± 5.7%. Void volume fraction decreased for all groups, as the injection into a confined space (e.g., gelatin gel or tissue) exerts a constant force on the hydrogel and influences packing. Yet, the rod‐like microgels still maintained a higher void volume fraction than equivalent volume spheres (Figure 4F). This highlights the importance of evaluating granular hydrogels in the context in which they are used.

### 2.5 Cell Invasion In Vivo Increases with Higher AR Rod‐Like Microgels

**Figure 5:**
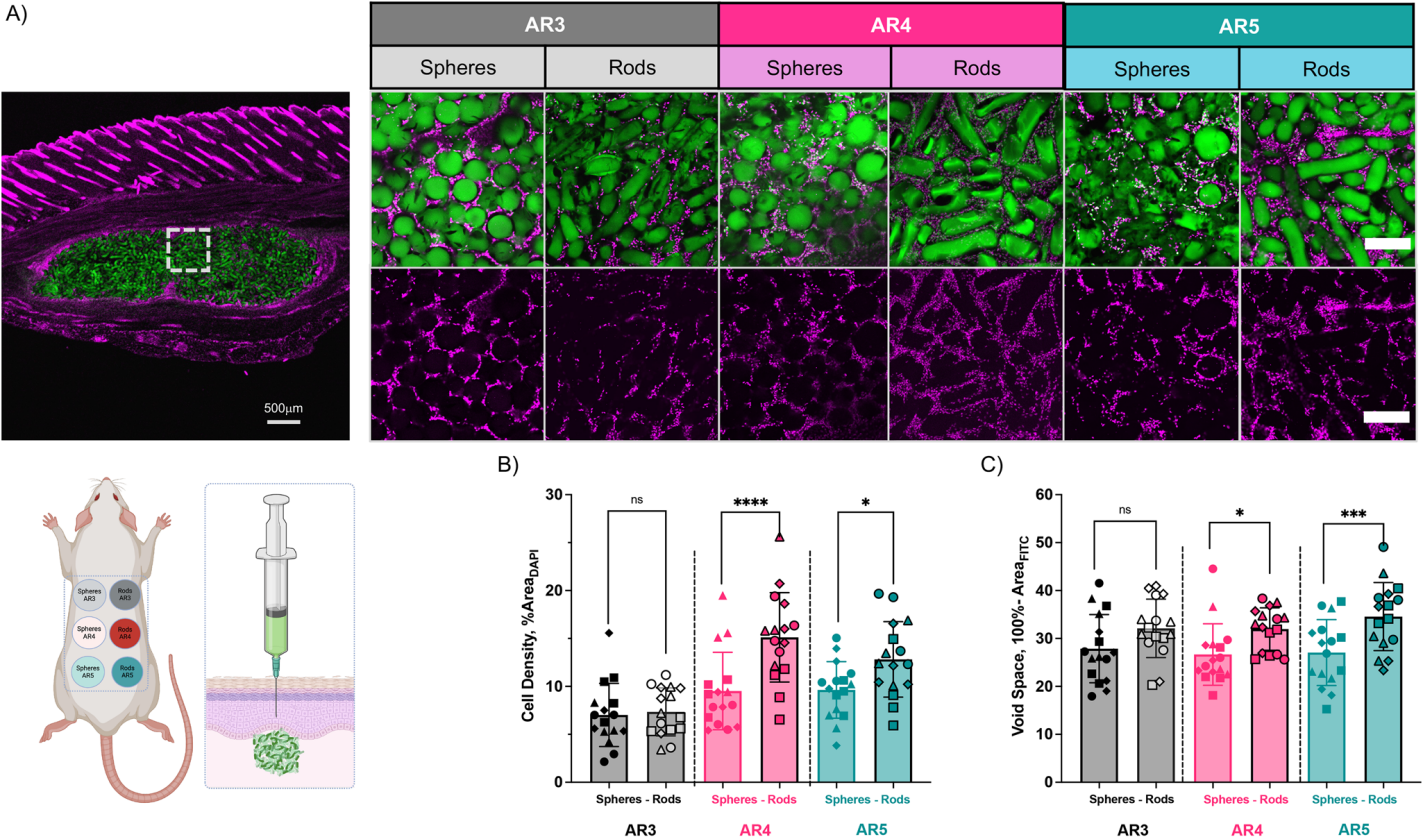
Cell invasion into granular hydrogels injected subcutaneously based on microgel shape and aspect ratio. A) Schematic of injection and representative confocal images of cell invasion into granular hydrogels after injection subcutaneously in rats (Cell nuclei: magenta, microgels: FITC‐Dextran). Scale bars = 200 µm. Quantification of B) cell density and C) void space between microgels 1 week after injection of granular hydrogels subcutaneously (*N* = 4 Implants X 4 Images Per Group; Each symbol (Circles, Squares, Triangles, Diamonds) denotes a specific rat). Mean (SD); ns: not s ignificant, *P *> 0.05; *P < 0.05; ***P < 0.001; ****P < 0.0001.

To assess granular hydrogel performance after injection, granular hydrogels were injected into the rat subcutis, and void space and cell invasion were evaluated at the scaffold‐tissue interface using confocal microscopy (**Figure** **5**A). This was performed across all groups, including granular hydrogels from rod‐like microgels of AR3, AR4, and AR5, as well as their volume‐matched spherical counterparts. Cell penetration was quantified using a 4',6‐diamidino‐2‐phenylindole (DAPI) stain for nuclei and quantifying within the granular hydrogel (Figure 5B), and void space was quantified by subtracting the area covered by microgels (via FITC as a fluorescence marker) from the total area (Figure 5C).

In general, granular hydrogels from higher AR rod‐like microgels exhibited increased void space and cell invasion when compared to spheres (Figure 5B,C). For example, the void space of AR5 rods was 34.6 ± 7.1% in comparison with the equivalent spheres at 27.1 ± 6.9% when measured in vivo. This anatomical location confirms the hypothesis of a higher void volume fraction achieved when using rod‐like microgels for scaffolds instead of spherical microgels, particularly with the higher AR formulations. Even without any cytokines or growth factors introduced, cell invasion was enhanced for the higher AR rod‐like microgels when compared to spherical microgels, and after one week, the percentage of cell‐covered area (DAPI) for AR5 rods was 12.8 ± 3.9% compared to the equivalent spheres at 9.64 ± 2.95%. Ultimately, there are numerous features that combine to result in increased cellular invasion in this environment, including void space (Figure 5C), pore size (Figure 2), and particle elongation (Figure 1C).

### 2.6 Granular Hydrogels from High AR Microgels Improve Repair after MI

To further demonstrate the capabilities of granular hydrogels from high AR microgels for clinical applications, we examined the capability of an acellular scaffold to mitigate cardiac remodeling and function post‐MI. As a power analysis using preliminary studies revealed that a large number of animals would be required to identify potential differences between spherical and rod‐like particles within this MI model, particularly due to potential variability in animal response, we chose to focus this work on illustrating the potential of injectable granular hydrogels from rod‐like microgels in such an application. Based on in vitro and in vivo results, rod‐like microgels with an AR5 were selected for the treatment group due to their enhanced void volume fraction, cell invasion, and ease of injectability. An ischemia‐reperfusion rat model was selected to evaluate the potential of the granular hydrogel and compared to an untreated MI control.

**Figure 6:**
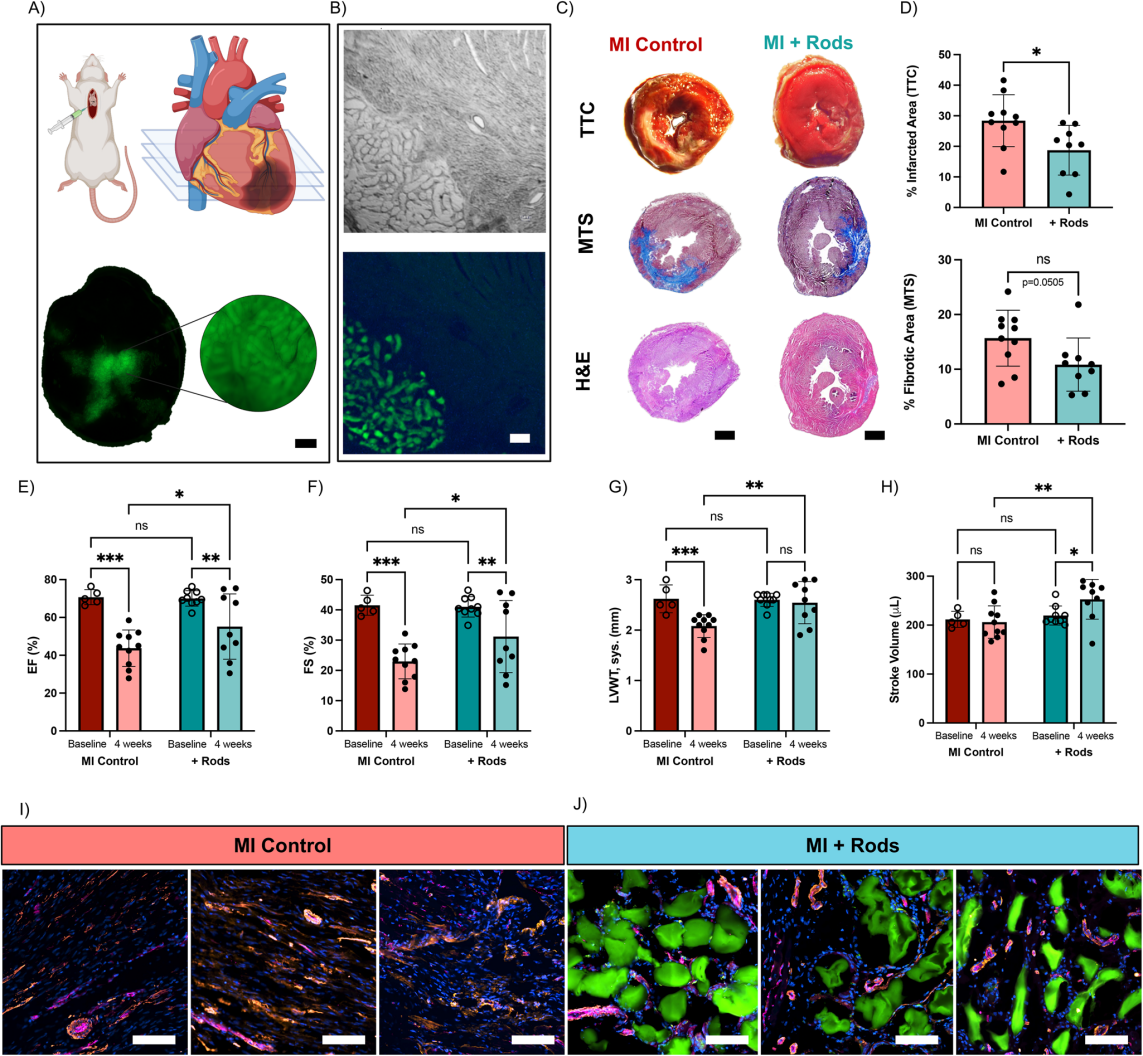
Influence of granular hydrogel injection on cardiac remodeling after infarction. A) Schematic of granular hydrogel injection (top) and fluorescence image of granular hydrogel formed from rod‐like microgels 4 weeks after injection (bottom). Scale bar = 200 µm. B) Brightfield (top) and fluorescence (bottom) images of a tissue section of granular hydrogel formed from rod‐like microgels 4 weeks after injection. Scale bar = 200 µm. C) Representative images of tissue sections and staining showing the infarcted (Triphenyl tetrazolium chloride (TTC) staining) and fibrotic (Masson's trichrome stain (MTS)) areas, as well as tissue architecture (Hematoxylin and eosin (H&E) stain). Scale bar = 2 mm. D) Quantification of infarcted and fibrotic areas identified by TTC (top) and MTS (bottom) staining. Echocardiography data showing the impact of MI and injection of rod‐like granular hydrogels on E) ejection fraction (EF), F) fractional shortening (FS), G) left ventricle wall thickness (LVWT) during systole, and H) stroke volume. Representative immunohistochemistry images of the infarcted area of I) untreated infarct controls and J) infarcts treated with rod‐like granular hydrogels (*n* = 3 rats). Tissue sections stained for cell nuclei (blue, DAPI), CD31 (magenta, AlexaFluor 647), aSMA (orange, AlexaFluor 555), and microgels (green, FITC). Blood vessels are identified when CD31 colocalizes with aSMA. Scale bars = 100 µm. The baseline was taken before the MI procedure. Mean (SD); ns: not significant, *p* > 0.05; *p < 0.05; **p < 0.01; ***p < 0.001.

Microscopy confirmed microgel retention in the heart until the end point of the study (**Figure** **6**A,B). After 4 weeks, the infarct size was quantified using triphenyl tetrazolium chloride (TTC) and the fibrotic area using Masson's trichrome stain (MTS) (Figure 6C,D; Figure S5, Supporting Information). Injection of rod‐like hydrogels reduced infarct size, likely due to hyaluronic acid interactions with fibroblasts and serving as an acellular filler (mechanical bulking) to reduce myocardial wall stress,^[^ ^59^ ^]^ as shown by TTC staining (+Rods 18.7 ± 8.1% vs Control 28.4 ± 8.5%). The decrease in the fibrotic area (MTS) between the control (15.2 ± 4.8%) and the granular hydrogel (10.7 ± 4.7%) was not significant (*p *= 0.0505). The microgels must also not restrict the heart beating to avoid deteriorating function for granular hydrogel injections to be effective.^[^ ^58^ ^]^ Previous injections of spherical annealed microgels did not show a negative impact,^[^ ^60^ ^]^ and no concerns were observed in this study. Furthermore, the modulus of the granular hydrogels was below 200 Pa (Figure 4A), which is orders of magnitude lower than the myocardium itself.^[^ ^61^ ^]^ Thus, the bulking of the myocardial wall should not adversely impact its function.

Cardiac function, monitored via echocardiography, showed significant improvements in ejection fraction (EF), fractional shortening (FS), and left ventricular wall thickness (LVWT) in the treatment group compared to MI controls (Figure 6E–G; Figure S6, Supporting Information). As a reference point, baseline measurements were taken prior MI. Notably, the differences in heart function at the baseline were not statistically significant, with an EF of 70.4 ± 0.5%. After MI, the EF decreased to 43.7 ± 9.6% for the control, with a more modest reduction to 55.2 ± 17.3% when the NorHA rod‐like microgels were injected. Both FS and LVWT also decreased after MI, but to a lesser extent when injected with the NorHA microgels. Increased stroke volume in rats treated with microgels over untreated MI controls (Figure 6F) may be from increased body weight during the four‐week period. Immunohistochemistry (IHC) and DAPI staining were then performed to assess cell invasion and angiogenesis. IHC identified CD31‐positive endothelial cells that co‐localized with αSMA in cylindrical‐like blood‐vessels in the infarcted areas treated with granular hydrogels. Mechanistically, this suggests that the intrinsic macroporosity of granular hydrogels improves cardiac function, in part by promoting angiogenesis within the scaffold while reducing wall stress.

## 3 Discussion

Granular hydrogels, with their intrinsic void space, offer a modular platform for scaffold fabrication. Our investigation focused on the impact of microgel shape and AR on void space, pore size, injectability, and cell invasion on acellular and non‐annealed scaffolds. Our study confirmed that non‐annealed, larger AR rod‐like microgels exhibited increased void volume fraction (53% to 73%) and pore size compared to volume‐matched spherical microgels when packed by centrifugation (25% to 46%). These results are within the range of other simulations and experimental work that has been shown before, with porosities ranging from 40% to 90% for ARs ranging from 2 to 20.^[^ ^6^, ^35^, ^36^, ^38^, ^40^ ^]^ However, we also showed that the injection process can greatly impact the behavior of granular materials.

One challenge in comparing results across studies is the differences in features such as microgel AR, the variability of jamming conditions used to pack the microgels, and variations in microgel stiffness and whether the microgels are annealed. For example, after fabrication and purification, microgels are jammed by applying pressure via vacuum or centrifugation forces,^[^ ^44^, ^62^ ^]^ and the extent of the force and the stiffness of the microgels impact microgel packing. Here, we used a high centrifugation speed (15 000 rcf) to pack the microgels, and previous reports confirm that increasing centrifugation speed (e.g., 1000 to 15 000 rcf) decreases the void volume fraction.^[^ ^6^ ^]^ Such trends have also been reported by others when packing spherical microgels by increasing the centrifugation force from 3000 to 16 000 rcf;^[^ ^62^ ^]^ however, differences in multiple parameters (e.g., microgel stiffness) make it challenging to compare absolute values across studies.

The De Laporte lab investigated rod‐like microgels with AR as high as 20,^[^ ^35^, ^40^ ^]^ which increased the void volume fraction compared to spherical microgels^[^ ^35^, ^40^ ^]^ but used a centrifugation speed of 4500 rpm.^[^ ^40^ ^]^ Stornerllo et al. observed no differences in void volume fractions (≈15%, lower than other reports) of granular hydrogels with rod‐like microgels with AR ranging 2 to 10, likely due to the use of microgels with a modulus of 17 kPa.^[^ ^41^ ^]^ In contrast, Suturin et al. reported a higher void volume fraction for AR 5 (≈65%) and AR 10 (≈75%) microgels, using more rigid microgels (modulus of ≈470 kPa) that were also annealed together.^[^ ^40^ ^]^ Although particle annealing is common, the kinetics of the annealing process may also be important in determining the void space, especially after injection. These two studies confirm an intuitive expectation that significant changes in the microgel modulus (from ≈17 up to 470 kPa) can have a substantial impact on void volume fractions (from 15% up to 75%) due to particle deformation and packing. The same holds true here. Due to recent studies from our group, we expect the stiffness of the 3 wt.% NorHA microgels to be ≈50 kPa.^[^ ^63^ ^]^ With compaction by centrifugation, we achieved a void volume fraction of up to 73% with higher AR rod‐like microgels, indicating limited deformation, as also observed under the microscope (Figure 2A). These values are within the magnitude observed by Suturin et al.^[^ ^40^ ^]^ When particles are under pressure for extrusion (Figure 4D) or constrained volumes (Figure 4E), there is a decrease in the void volume fraction, comparable to the magnitudes reported by Stornerllo et al.^[^ ^41^ ^]^ Still, the void volume fraction of granular hydrogels from rod‐like microgels (from 13 to 24%) was higher than the equivalent spherical microgels (from 5% to 11%). This observation further supports one of the objectives of our work, highlighting the impact of AR in granular hydrogel design.

Another major challenge when comparing structures across studies for granular hydrogels is the use of methods that may not encompass the whole story, such as two‐dimensional (2D) images that may be limited toward a 3D understanding, particularly with non‐spherical microgels. Using LOVAMAP software, we characterized the end‐to‐end 3D pore length and average internal diameter and demonstrated how the shape affected the pore dimensions, which is critical for cell invasion. The median internal 3D pore diameter for the rod‐like microgels was above 50 microns, within the range previously shown to enhance vascularization on porous bulk hydrogel models.^[^ ^15^, ^16^ ^]^ Even though other studies have demonstrated successful cell invasion on spherical microgel scaffolds of smaller pore sizes,^[^ ^11^ ^]^ here we show a significant improvement for rod‐like microgels compared to spherical microgel scaffolds. In vitro and in vivo assessments demonstrated higher cell invasion for higher AR microgels. These wider and longer pores might be a contributing factor to a reduced path tortuosity for cell infiltration, as seen in the in vitro sprouting assay. Differences in the shape and dimensions of microgels also have an impact on the curvature of the microgels and resulting pores when jammed into granular hydrogels. Previous research has found that surface curvature impacts cell migration;^[^ ^64^, ^65^ ^]^ thus, it is a potential consideration in granular hydrogels since all surfaces exhibit curvature.

Overall, rod‐like microgels provided adequate pore size and enhanced void volume fraction that resulted in increased sprouting displacement and cell invasion. From an engineering perspective, injecting these non‐annealed microgels would be advantageous for delivery and allow microgel rearrangements in dynamic tissues like the heart. For other scenarios, such as open wound healing, annealing of microgels would be advantageous.^[^ ^4^ ^]^ However, the same design principles discussed in this study would apply since microgels could be annealed after injection. In this work, we prepared granular hydrogels from microgels that had a single shape. By combining particles of different geometries, such as incorporating rod‐like microgels into a spherical granular scaffold, there are even additional options for tuning the pore space and scaffold properties. Computer simulations have been used to study the impact of polydispersity on pore space by combining spherical microgels of different diameters.^[^ ^38^ ^]^ However, the combination of microgels of different shapes, to the best of our knowledge, remains relatively unexplored.

This study demonstrates how shape and AR of microgels impact granular hydrogel design for injectable applications and illustrates how this knowledge can be used for tissue repair. To assess how this injectable granular scaffold would impact tissue remodeling and function, we utilized an ischemia‐reperfusion MI model combined with echocardiography and immunohistochemistry to evaluate the scaffold's impact. Based on the above findings, we selected rod‐like microgels at a high AR5 as the treatment group to assess the impact on cardiac remodeling. Injecting rod‐like microgels immediately post‐ischemia minimized surgical procedures, although injection a few days after injury might be closer to eventual translational practices. After four weeks, microgel retention, distribution, and cell invasion in the myocardium were confirmed using confocal microscopy. Rod‐like microgels reduced infarct size and fibrosis and promoted angiogenesis, supporting the theoretical advantages of granular hydrogels. Previous work has established that hydrogel injections reduce the peak myocardial strain and suggested that they contribute to reducing infarct expansion.^[^ ^59^ ^]^ Based on that, it is likely that the viscoelastic properties of a granular hydrogel could contribute to the wall stress dissipation over the cardiac cycle while bulking the myocardium.

Although this cardiac study did not address any questions related to microgel shape, it does expand the use of granular hydrogels in tissue repair. Also, microgels were fabricated using NorHA, which leverages the potential bioactivity of HA for cardiac repair.^[^ ^66^, ^67^, ^68^, ^69^ ^]^ Previous studies demonstrated the potential of HA hydrogels, after injection in the heart, but these non‐porous structures did not leverage the benefits of granular hydrogels. As an example of how this system could be further tuned, HA molecular weight (MW) has been shown to impact tissue response and angiogenic potential of HA hydrogel scaffolds.^[^ ^70^, ^71^, ^72^, ^73^ ^]^ For this study, we used a 70 kDa HA for the fabrication of all microgels, but even higher MWs could be utilized for microgel fabrication. Another group found that spherical microgels of 1 MDa HA promoted higher vessel formation than for those from 40 kDa HA in a subcutaneous rat model.^[^ ^72^ ^]^ Although HA micro‐rods have been used previously to treat myocardial infarction,^[^ ^67^, ^74^ ^]^ they have been used as a delivery mechanism, not as a granular scaffold where the particles are in contact. In one study, the micro‐rods were mainly an HA delivery mechanism instead of bulk gels, and the anti‐fibrotic effects were characterized both in vitro and in vivo.^[^ ^67^ ^]^ In a second study, the micro‐rods were loaded with anti‐fibrotic proteoglycan decorin to attenuate cardiac fibrosis further.^[^ ^60^ ^]^ In both cases, these micro‐rods were diluted in saline, acting as suspended microgels and not as a granular scaffold. Although the objectives of those studies are different, they provide complementary input into the mechanical effects of drug‐loaded microgels that could be incorporated into the design.

The outcomes of this study regarding microgel AR and influence on microgel packing, injectability, and cell invasion extend to other scaffold fabrication applications, potentially leading to broad advances in the design of materials as patient therapies. The successful outcome of rod‐like microgels offers promising steps toward tissue engineering, and this was illustrated here for cardiac tissue repair strategies. Finally, although we engineer an acellular granular scaffold using HA, other chemistries, cytokines, or drugs could also be introduced during microgel fabrication to enhance post‐infarct remodeling or other biological outcomes.

## 4 Experimental Section

### Microgel Fabrication and Characterization–Materials

All materials were purchased from Sigma Aldrich unless specified otherwise.

### Hyaluronic Acid Functionalization

Norbornene‐modified hyaluronic acid (NorHA) was synthesized as described in previous work.^[^ ^75^ ^]^ Briefly, sodium ions of the hyaluronic acid salt (HA, Lifecore, 70 kDa) were exchanged into a tetrabutylammonium (TBA) group via a two‐step process, first using a Dowex 50 W × 200 resin and then a tetrabutylammonium hydroxide solution. The HA‐TBA was dissolved in dimethyl sulfoxide (DMSO) to react with 5‐norbornene‐2‐carboxylic acid (Nor) using di‐tertbutyl dicarbonate (Boc2O) as a coupling agent and 4‐dimethylamine pyridine (DMAP) as a catalyst. Functionalization was confirmed and quantified using ^1^H NMR (Figure S7, Supporting Information).

### Fabrication of Rod‐Like Microgels

Rod‐like microgels were fabricated using polydimethylsiloxane (PDMS) based single‐channel flow focusing microfluidic droplet generators with a constricted channel cross‐section designed to squeeze droplets generated into a rod shape, similar to the method previously used to make rod‐like microgels of AR 2.2. The dispersed phase in this system was 30 mg mL^−1^ NorHA, 0.8 v/v% dithiothreitol (DTT), and 0.075 w/v% Lithium phenyl‐2,4,6‐trimethylbenzoylphosphinate (LAP) photoinitiator, and the continuous phase in the system was HFE (3 M Novec 7500) with 2 wt.% Krytox (Miller‐Stephenson, Cat No.157 FSH). FITC‐Dextran 2 MDa 0.2 w/v% was added to visualize microgels. The devices were silanized using trichloro(1H, 1H, 2H, 2H‐perfluorooctyl) silane. The dispersed (*ɸ*_D_) and continuous (*ɸ*_C_) phase flow rates were changed to produce droplets of diameter (*d*_d_) = 120, 140, and 160 µm. Droplet size was determined via image analysis of microscope images of the droplets exiting the device outlet, without being exposed to UV. Typically, the initial flow rates used to produce droplets of diameter *d*_d_ = 120 µm were *ɸ*_D _= 0.15 mL h^−1^ and *ɸ*_C_ = 1 mL h^−1^; to produce droplets of diameter *d*_d_ = 140 µm were *ɸ*_D _= 0.3 mL h^−1^ and *ɸ*_C_ = 0.7 mL h^−1^; to produce droplets of diameter *d_d_* = 160 µm were *ɸ*_D_ = 0.4 mL h^−1^ and ɸ_C_ = 0.6 mL h^−1^; however, the flowrates were further tuned each run, to better fit the desired aspect ratio on‐chip in order to account for batch to batch variation. Droplets with *d*_d_ = 120, 140, and 160 µm were all squeezed through a channel cross‐section of 100 µm × 100 µm, resulting in rod‐like droplets of different aspect ratios.

All rod‐like microgels were exposed to a 405 nm laser at ≈15.1 W cm^−2^ power (P) on chip (measured using a Thorlabs S415C thermal power sensor head, and a PM100D power meter console), and then were further exposed to 365 nm UV light using a BLAK‐RAY LAMP (Longwave UV‐366 nm). Total flow rate (*ɸ*_T_) was controlled in order to alter the UV dosage to crosslink the NorHA within rod‐like hydrogels. Droplets (120 µm) were exposed to a UV dosage of 1.2 × 10^−3^ W‐s on chip, resulting in rod‐like microgels of AR 3 (*ɸ*_T_ = 1.15 mL h^−1^). Larger (*d *= 140 µm, *d *= 160 µm) droplets were exposed to a UV dose of 1.4 × 10^−3^ W‐s, resulting in rod‐like microgels of AR 4 and 5 (*ɸ*_T_ = 1 mL h^−1^). UV dosage was calculated using the formula: dosage = (𝑃*x*𝐴)/𝑡_𝐸_, where the illumination area (*A*) was measured to be 0.0016 cm,^[^ ^2^ ^]^ and the exposure time (*t*_E_) for AR3 was calculated to be 0.05s, while the exposure time for AR4 was calculated to be 0.0576 s. The required on‐chip UV dosage was determined experimentally as the UV exposure required by the rods to maintain their shape without deforming or merging until collection. All rods were collected and further cured using the off‐chip UV light source to equalize the total UV exposure between different AR rods.

The rod‐like microgels in HFE‐7500 were collected into 1.7 mL Eppendorf tubes containing 20 vol% of 1H, 1H, 2H, 2H‐perfluoro‐1‐octanol to destabilize the surfactants. After centrifugation, the majority of the oil was removed. The microgels were then washed sequentially with hexane, a 50:50 mixture of ethanol, and PBS to remove the remaining oil. Between each step, a centrifugation at 9700 relative centrifugal force (rcf) for 1 min was performed.

### Fabrication of Spherical Microgels

To form spherical microgels, a simple single‐channel flow‐focusing microfluidic device was utilized.^[^ ^6^ ^]^ This PDMS device was formed using a 3D‐printed master mold to create 100 µm channels for aqueous and continuous phases that then connect at a 300 µm channel for droplet generation. Inlet and outlet channels were created using 1 mm biopsy punches. The device was plasma‐bonded to a glass slide, and blunt stainless steel needles were inserted into the inlet and outlet channels.

Spherical microgels were fabricated as described previously.^[^ ^5^ ^]^ Briefly, the dispersed phase in this system was 30 mg mL^−1^ NorHA, 0.8 v/v% DTT, and 0.1 w/v% LAP. FITC‐Dextran 2 MDa 0.2 w/v% was added to visualize microgels. The syringe containing the aqueous phase was protected from light to avoid pre‐polymerization. Flow rates for both phases were optimized to obtain stable droplet generation and to change droplet size. The aqueous dispersed phase flow rate (*ɸ*_D_) ranged from 0.08 to 0.15 mL h^−1^, while the continuous oil phase flow rate (*ɸ*_C_) was adjusted from 0.8 to 1.2 mL h^−1^. The outlet tube was configured into a spiral and irradiated with a light source (UV filter 320–390 nm) set at 20 mW cm^−2^ to crosslink the polymer off‐chip before flowing into a collection tube. It is important to note that the flow rates were adjusted to obtain microgels of different diameters, matching the volume of rod‐like microgels. Spherical microgels were then centrifuged and purified as described previously.^[^ ^5^ ^]^

### Quantification of Microgel Dimensions

Following the fabrication of microgels, a 2 µL aliquot of microgels was placed on a glass slide and imaged using a widefield microscope, with at least three different images taken of the microgels. Using ImageJ software, the diameter of ten microgels was measured on each image. For rod‐like microgels, the diameters and lengths of ten microgels were measured to determine the average aspect ratio of each image.

### Granular Hydrogel Fabrication and Characterization–Cinema 4D Void Volume Fraction Simulations

Various AR rod‐like and corresponding volume‐matched spherical microgel packing was simulated (Cinema 4D S24.111) using rigid‐body dynamics and processed in Slicer 5.2.1 and FIJI to obtain % void volume fraction measurements. Rod and spherical microgels were modeled as a capsule (Radius: *R*, Height: *H*) and a sphere (R) rigid mesh bodies with dimensions identical to the mean radius and height of experimental microgel measurements. An open cylinder with the dimensions *R* = 1mm and *H* = 1 mm was created to collect microgels as they packed via simulated gravity. Each microgel was given a dynamic body tag to enable Newtonian physics, and the open cylinder was given a collider body tag to negate the force of gravity and to capture packing microgels. Each simulation was run for 725 frames at 30 frames per second (FPS), at the final frame was then baked and exported as an.stl for processing. To add variability into the model, each starting microgel “seed” position point was randomized for every granular hydrogel replicate simulated (*n* = 3). Once the.stl file was created for the given simulation, the triangulated mesh body was imported into Slicer 5.2.1, sliced to obtain a constant voxel height of 10 µm (100 slices), and thresholded to obtain outlines of the granular hydrogel simulations. The minimum microgel feature for these simulations was 129.4 µm in diameter and thus, a voxel height of 10 µm was sufficient to capture all features. The interior of granular hydrogel slices was filled such that all pixels outside of microgels were set to zero. Slicer 5.2.1 granular hydrogel slices were then exported as a.tiff image and imported into FIJI as a z‐stack, thresholded (via default) for pore spaces, and the voxel setting adjusted to mimic the original dimensions. Each simulated z‐stack was processed via a 3D object counter^[^ ^76^ ^]^ to obtain a volume for the thresholded pore fraction, which was presented as % void volume fraction and calculated based on the theoretical volume of the filled cylinder. Images of the granular hydrogel scaffolds were captured in a central region to mitigate border effects.

### Granular Hydrogel Fabrication and Quantification of Void Volume Fraction

Granular hydrogels were fabricated by compacting the suspended microgels by centrifugation at 15 000 rcf for 5 min. The supernatant was decanted, and the granular hydrogel pellet was transferred for further use with a wide‐bore pipet tip. Microgels were loaded with FITC‐dextran 2 MDa during fabrication to visualize and quantify void volume fraction by confocal microscopy (Nikon Eclipse Ti2 inverted microscope, 20X immersion objective lens). The region of interest (ROI) for the bulk void volume fraction was 778 × 778 × 600 µm with a z‐step size of 2 µm. For each group, 3 independent scaffolds were imaged in two locations in the center of the sample, avoiding border effects.

Using this approach, the in vitro void volume fraction was also quantified after extrusion through a syringe. Microgels were injected into bulk gelatin hydrogel (1.5 wt.%) blocks of 25 × 25 × 1.5 mm dimensions. The gelatin blocks were cured between a glass slide and a cover slip to allow visualization on the confocal microscope. The ROI for the hydrogels injected into the gelatin hydrogels was pockets of granular hydrogels of 707 × 707 × 250 µm with a z‐step size of 2 µm. Using the threshold function in ImageJ, all stacks were binarized, and then a macro used to compute the void volume fraction throughout the sample.

### Analysis of Scaffold Pores

LOVAMAP software was used to identify and study the 3D pores within the scaffolds.^[^ ^17^ ^]^ A particle segmentation approach as reported in Riley et al.^[^ ^38^ ^]^ was first used with confocal imaging data. The custom code utilized open‐source Python libraries, such as numpy, scikit‐image, and scipy, to interpolate across binarized z‐stacks of fluorescently‐labeled particle scaffolds to identify unique particles using a watershed‐based methodology. The particle‐segmentation data were then fed into LOVAMAP, where particle location was used to identify medial axis landmarks that guide 3D pore segmentation. 3D pores represent the natural open pockets of void space between packed particles. Since the microscope images are a subspace of a larger granular hydrogel scaffold, particles – and therefore 3D pores – were truncated at the edge of the samples. 3D pores that lie along the edge of the sample were referred to as “surface pores,” while pores entirely encased within the scaffold were referred to as “interior pores.” LOVAMAP was used to report the scaffold void volume fraction, as well as 3D pore metrics described in Riley L, et al.^[^ ^17^, ^53^ ^]^

### Granular Hydrogel Mechanical Characterization

A strain sweep was performed using a Discovery Hybrid Rheometer HR‐20 from 0.1 to 100% strain at 0.5 Hz. Granular hydrogels were loaded into 8 mm parallel plates at a 1 mm gap. To quantify the injection force, hydrogels were loaded into a 1 mL syringe with a 27‐gauge needle (1.5 inches in length). The syringe was then placed into a syringe pump with an Arduino‐based force sensor setup, as described elsewhere.^[^ ^44^ ^]^ Force measurements were taken by injecting ≈400 µL of microgels at a flow rate of 20 µL s^−1^.

### Investigation of Cellular Invasion into Granular Hydrogels–In Vitro Cellular Sprouting Assay

An assay to measure cellular migration and sprouting from spheroids embedded in granular hydrogels was used as previously described.^[^ ^44^ ^]^ Briefly, spheroids of endothelial/mesenchymal cells (2:1 ratio, ≈1000 cells spheroid^−1^) were prepared with AggreWell 400 templated agarose wells). Individual spheroids were pipetted into the granular materials scaffolds fabricated using 50 µL of microgels (centrifuged at 15 000 rcf, 5 min.). The interstitial space contained 10 mg mL^−1^ NorHA, a thiolated RGD sequence (1 mm, GCGYGRGDSPG, Genscript), a thiolated matrix metalloproteinase (MMP) degradable sequence (3 mm, GCNSVPMSMRGGSNCG, Genscript), and LAP photoinitiator (0.05w/v%). The interstitial matrix was crosslinked by exposing the scaffold to a UV light source (320–390 nm filter, 20 mW cm^−2^) for 2 min. Scaffolds were rinsed with PBS twice to remove any unreacted species. Spheroids were cultured for 3 days using media supplemented with vascular endothelial growth factor (VEGF, 100 ng mL^−1^), sphingosine‐1‐phosphate (S1P, 500 nm), and phorbol 12‐myristate 13‐acetate (PMA, 600 ng mL^−1^) to assess cell sprouting into the granular material. Cells were fixed, stained with Alexa Fluor 647 Phalloidin, and imaged using confocal microscopy. Sprout length, displacement, and tortuosity were determined using the Manual Tracking option in ImageJ.

### Subcutaneous Invasion Model

Subcutaneous rat studies were approved by the Institutional Animal Care and Use Committee at the University of Colorado Boulder (approval number: 0 3009). Under general anesthesia using 2–4% isoflurane, ten‐week‐old male Wistar rats (Charles River) weighing ≈300 g were shaved around their dorsum. After three alternate scrubs using chlorhexidine and 70% ethanol, rats (*n* = 4) were subcutaneously injected with 200 µL of a granular hydrogel. Each rat was injected at 6 different locations (randomized across rats) with granular hydrogels from rods of AR 3, 4, and 5, as well as spheres of equivalent volumes. After 1 week, rats were humanely euthanized, and circular biopsies that included the pouch containing samples and surrounding tissues were collected. Tissues were fixed in formaldehyde for 24 h, submerged in 15% sucrose for 24 h, and then 30% sucrose for another 24 h. The tissues were embedded in optical cutting temperature (OCT) compound and frozen at −80 °C. The tissues were sectioned on a cryostat (thickness 40 µm). Cells were stained with DAPI for 5 min at room temperature and imaged with a Nikon Eclipse Ti2 inverted microscope with a 20× immersion objective lens. ImageJ's built‐in functions and plugins were used to quantify the area and assign colors. Using the threshold function, the stack(s) were binarized and then used a macro to compute the cross‐sectional area of microgels and cell nuclei (DAPI) on each image.

### Granular Hydrogel Therapeutics for Myocardial Infarction–Myocardial Infarction Model

For myocardial infarction (MI), an ischemia‐reperfusion injury model approved by the Institutional Animal Care and Use Committee at the University of Colorado Boulder (approval number: 2871) was used. Similar to subcutaneous studies, 10–12 week‐old male Wistar rats (Charles River) weighing ≈300 g were used. Animals received a single subcutaneous injection of sustained‐release buprenorphine (1.2 mg kg^−1^) as preoperative analgesia. Afterward, animals were induced in 5% isoflurane before being intubated intratracheally for mechanical ventilation using 2% isoflurane. After surgical scrub, a left thoracotomy at the 4th intercostal space was performed, with lidocaine infiltration in the intercostal muscles. Following the placement of retractors, the left anterior descending (LAD) coronary artery was identified and ligated using a 6‐0 silk suture tied around a sterile monofilament nylon suture (LOOK 1, Hospeq Medical Equipment and Supplies). A major inclusion criterium for this study was successful ligation of the LAD, confirmed by tissue blanching distal to the suture. The chest retractors were removed, and the skin was temporarily apposed, while the animal remained ventilated during the 30‐minute ligation. Afterward, the ligature around the LAD was removed for reperfusion, which could be seen by the return of color to the myocardial tissue.

Rats were assigned to one of 2 groups: MI controls (no intramyocardial injections; *n* = 10) or MI + rods (received 5 intramyocardial injections totaling 100 µL using a 27G needle; *n* = 9). Rats without successful intramyocardial injection, where backflow of injected hydrogels resulted in a lack of retention, were excluded from the study. The intercostal muscles and ribs were closed with a simple interrupted pattern using a 4‐0 prolene suture, followed by evacuation of air and fluid from the thoracic cavity via a 16G angiocatheter connected to a 10 mL syringe to establish normal pleural negative pressure. Separately, the pectoral muscle and skin were closed with a simple continuous suture pattern using a 4‐0 polyglactin 910 (Vicryl) ligature. In addition to postoperative care, animals received prewarmed Lactated Ringers (5 mL) and meloxicam 2 mg kg^−1^; postoperative analgesia lasted 3 days (once daily). Four weeks post‐surgery, non‐invasive measurements of left ventricular (LV) dimensions and function were obtained using a Vevo 3100 ultrasound imaging system (Visual Sonics). Note that, as a reference point, baseline measurements were also taken before MI at Day 0. Under general anesthesia (2% isoflurane), the surgical site was shaved and depilatory cream (Nair) applied before echocardiography to obtain measurements. Afterward, rats were humanely euthanized and heart tissues collected for analyses.

### Tissue Analysis

Four weeks after infarction, hearts were frozen for 30 min at −20 °C in freezing paper and then sectioned into 3 mm sections. The sections were stained with a PBS solution of 1 wt.% 2,3,5‐Triphenyltetrazolium chloride (TTC) for 30 min, fixed in formaldehyde, and imaged using a Nikon stereoscope. The infarcted area was quantified on the main ventricle sections, disregarding the apex and sections above the heart valves. Image J's hand wand tool was used to quantify the left ventricle and infarcted areas.

Heart sections were fixed in formaldehyde for 24 h, submerged in 15% sucrose for 24 h, and then 30% sucrose for another 24 h. The hearts were embedded in OCT and sectioned on a cryostat (thickness 10 µm). Heart sections were stained using Masson's Trichrome and H&E to visualize the infarcted region and fibrotic tissue. Masson's Trichrome Stain Kit was purchased from Polysciences (No. 25088‐1). Chemicals for H&E staining were purchased from Sigma. Masson's Trichrome staining defined the infarct size as the ratio of the fibrotic area (blue stain) to the left ventricle (LV) area. The image was quantified using ImageJ and converted to an HSB file to allow the wand tool to quickly and accurately select the blue‐stained area of the heart sections.

Cryosections (thickness 30 µm) from the infarcted area of each heart were stained by immunochemistry to identify CD31^+^ and  αSMA^+^ cells. Slides were heat‐treated by submersion on 10 mm sodium citrate with buffer, pH 6.0 (≈90 °C for 5 min). After cooling down, a 5% FBS solution was used as a blocking agent. PBS was used for rinsing between steps. Sections were allowed to react for 90 min at room temperature with primary antibodies against CD31 (Novus, No. NB100‐2284) and αSMA (R&D Systems, No. MAB1420‐SP). Sections were subsequently stained with secondary antibodies conjugated with Alexa Fluor 647 (ThermoFisher No. A31573) and Alexa Fluor 555 (Invitrogen, No. A32727) for 90 min at room temperature. Cells were stained with DAPI for 5 min at room temperature and imaged with a Nikon Eclipse Ti2 inverted microscope with a 20× immersion objective lens at a 2× magnification.

### Statistical Analysis

Statistical analysis was performed using GraphPad Prism version 9.0.2 at the significance level of *p* < 0.05. Microgels and granular scaffolds were characterized by showing all individual values, means, and standard deviations. The normality of the data was assessed using the Kolmogorov–Smirnov test and the Shapiro–Wilk test. Statistical comparisons were made using analysis of variance (ANOVA) for multiple comparisons with the Tukey post‐hoc test. The Tukey post‐hoc test compared all possible pairs of means based on a studentized range distribution (q). Pore descriptor data generated by LOVAMAP was analyzed using the Kruskal–Wallis test (one‐way ANOVA on ranks). The Kruskal–Wallis test is the default on Prism for a nonparametric test to determine if there are statistically significant differences between two or more groups using the one‐way ANOVA analysis tool. Two‐way ANOVA was used to assess the impact of both the shape factor (spheres vs rods) and the AR factor. This analysis used the Sidak multiple comparisons test with a single pooled variance. The fibrotic area of the control and treatment groups was compared using an unpaired *t‐*test (two‐tailed). The echocardiogram data were analyzed using a two‐way ANOVA mixed model. When fitting the mixed model in GraphPad, the software considered that the data were matched by treatment (control, +rods) and time points collected for each group (baseline, four weeks).

### Code Availability

Details about the LOVAMAP code were published elsewhere.^[^ ^17^ ^]^ Access to the code is available upon request to Duke University. The copyrights of the LOVAMAP code are owned by Duke University. To request a license for the code, please send an email to otcquestions@duke.edu with reference to ‘OTC File No. 7784′. CC BY‐NC‐SA licenses for the code are free for academics.

## Conflict of Interest

The authors declare no conflict of interest.

## Author Contributions

G.J.R.R. and J.A.B. designed the project. G.J.R.R., S.S., and S.B. synthesized and characterized the microgels. G.J.R.R. performed sprouting assays. N.D. conducted simulations on particle packing. L.R. and C.E.M. conducted computational analysis of scaffold pores. C.M. and A.R.P. developed the MI model and performed in vivo studies. G.J.R.R., C.M., and S.B. stained and analyzed tissues. D.I., D.L., T.S., and J.A.B. supervised the project. G.J.R.R., C.M., S.S., S.B., N.D., L.R., and J.A.B. wrote the manuscript. G.J.R.R., D.I., T.S., and J.A.B. revised the manuscript. All authors discussed results, interpreted the data, and provided input on the paper.

## Supporting information



Supporting Information

## Data Availability

All data generated or analyzed during this study are included within this article, its Supporting Information, and its Source Data. Additional information is available from the corresponding author on request. Source data are provided with this paper.

## References

[1] A. C. Daly , M. D. Davidson , J. A. Burdick , Nat. Commun. 2021, 12, 753. 33531489 10.1038/s41467-021-21029-2 PMC7854667

[2] A. C. Daly , L. Riley , T. Segura , J. A. Burdick , Nat. Rev. Mater. 2020, 5, 20. 34123409 10.1038/s41578-019-0148-6 PMC8191408

[3] D. L. Elbert , Acta Biomater. 2011, 7, 31. 20659596 10.1016/j.actbio.2010.07.028 PMC2967636

[4] D. R. Griffin , W. M. Weaver , P. O. Scumpia , D. Di Carlo , T. Segura , Nat. Mater. 2015, 14, 737. 26030305 10.1038/nmat4294 PMC4615579

[5] V. G. Muir , T. H. Qazi , J. Shan , J. Groll , J. A. Burdick , ACS Biomater. Sci. Eng. 2021, 7, 4269. 33591726 10.1021/acsbiomaterials.0c01612 PMC8966052

[6] T. H. Qazi , J. Wu , V. G. Muir , S. Weintraub , S. E. Gullbrand , D. Lee , D. Issadore , J. A. Burdick , Adv. Mater. 2022, 34, 2109194. 10.1002/adma.202109194 PMC8957565 34932833

[7] T. H. Qazi , J. A. Burdick , Biomater. Biosyst. 2021, 1, 100008. 36825161 10.1016/j.bbiosy.2021.100008 PMC9934473

[8] N. D. Caprio , M. D. Davidson , A. C. Daly , J. A. Burdick , Adv. Mater. 2024, 36, 2312226. 10.1002/adma.202312226 PMC10994732 38178647

[9] B. A. Aguado , W. Mulyasasmita , J. Su , K. J. Lampe , S. C. Heilshorn , Tissue Eng., Part A 2011, 18, 806. 22011213 10.1089/ten.tea.2011.0391 PMC3313609

[10] A. C. Daly , Adv. Healthcare Mater. 2024, 13, 2301388.

[11] Z. Ataie , S. Horchler , A. Jaberi , S. V. Koduru , J. C. El‐Mallah , M. Sun , S. Kheirabadi , A. Kedzierski , A. Risbud , A. R. A. E. Silva , D. J. Ranvic , A. Sheikhi , Small 2024, 20, 2307928. 10.1002/smll.202307928 PMC11699544 37824280

[12] A. R. Anderson , E. Nicklow , T. Segura , Acta Biomater. 2022, 150, 111. 35917913 10.1016/j.actbio.2022.07.051 PMC10329855

[13] A. E. Widener , A. Roberts , E. A. Phelps , Adv. Healthcare Mater. 2004, 13, 2303005. 10.1002/adhm.202303005 PMC11196388 38145369

[14] T. H. Qazi , D. J. Mooney , G. N. Duda , S. Geissler , Biomaterials 2017, 140, 103. 28644976 10.1016/j.biomaterials.2017.06.019

[15] Y.‐C. Chiu , M.‐H. Cheng , H. Engel , S.‐W. Kao , J. C. Larson , S. Gupta , E. M. Brey , Biomaterials 2011, 32, 6045. 21663958 10.1016/j.biomaterials.2011.04.066

[16] H. Li , K. S. Iyer , L. Bao , J. Zhai , J. J. Li , Adv. Healthcare Mater. 2024, 13, 2301597. 10.1002/adhm.202301597 37499268

[17] L. Riley , P. Cheng , T. Segura , Nat. Comput. Sci. 2023, 3, 975. 38177603 10.1038/s43588-023-00551-x

[18] M. S. Flores‐Jiménez , A. Garcia‐Gonzalez , R. Q. Fuentes‐Aguilar , ACS Appl. Bio Mater. 2023, 6, 1. 10.1021/acsabm.2c00740 36599046

[19] T. M. S. Udenni Gunathilake , Y. C. Ching , K. Y. Ching , C. H. Chuah , L. C. Abdullah , Polymers 2017, 9, 160. 30970839 10.3390/polym9050160 PMC6431923

[20] P. yadav , G. Beniwal , K. K. Saxena , Mater. Today: Proc. 2021, 44, 2623.

[21] J. Rnjak‐Kovacina , S. G. Wise , Z. Li , P. K. M. Maitz , C. J. Young , Y. Wang , A. S. Weiss , Biomaterials 2011, 32, 6729. 21683438 10.1016/j.biomaterials.2011.05.065

[22] B. B. Mandal , S. C. Kundu , Biomaterials 2009, 30, 2956. 19249094 10.1016/j.biomaterials.2009.02.006

[23] W. Li , F. Dai , S. Zhang , F. Xu , Z. Xu , S. Liao , L. Zeng , L. Song , F. Ai , ACS Appl. Mater. Interfaces 2022, 14, 20693. 35500207 10.1021/acsami.2c02001

[24] Y. Zhang , N. Sun , M. Zhu , Q. Qiu , P. Zhao , C. Zheng , Q. Bai , Q. Zeng , T. Lu , Biomater. Adv. 2022, 133, 112651. 35034817 10.1016/j.msec.2022.112651

[25] J. M. Ameer , A. K. Pr , N. Kasoju , J. Funct. Biomater. 2019, 10, 30. 31324062 10.3390/jfb10030030 PMC6787600

[26] U. Stachewicz , P. K. Szewczyk , A. Kruk , A. H. Barber , A. Czyrska‐Filemonowicz , Mater. Sci. Eng., C 2019, 95, 397. 10.1016/j.msec.2017.08.076 30573264

[27] Y. Zhang , M. Zhang , D. Cheng , S. Xu , C. Du , L. Xie , W. Zhao , Biomater. Sci. 2022, 10, 1423. 35170597 10.1039/d1bm01651b

[28] Y. S. Zhang , C. Zhu , Y. Xia , Adv. Mater. 2017, 29, 1701115. 10.1002/adma.201701115 PMC5581229 28649794

[29] B. Markicevic , Powder Technol. 2019, 350, 154.

[30] Y. Ma , X. Wang , T. Su , F. Lu , Q. Chang , J. Gao , Gels 2022, 8, 606. 36286107 10.3390/gels8100606 PMC9601978

[31] C. M. Murphy , F. J. O'Brien , Cell Adhes. Migr. 2010, 4, 377. 10.4161/cam.4.3.11747 PMC2958613 20421733

[32] L. Riley , L. Schirmer , T. Segura , Curr. Opin. Biotechnol. 2019, 60, 1. 30481603 10.1016/j.copbio.2018.11.001 PMC6534490

[33] R.‐C. Tang , L. Shang , P. O. Scumpia , D. Di Carlo , Adv. Healthcare Mater. 2024, 13, 2302477. 10.1002/adhm.202302477 PMC11102933 37985462

[34] C. Tuftee , E. Alsberg , I. T. Ozbolat , M. Rizwan , Trends Biotechnol. 2024, 42, 339. 37852853 10.1016/j.tibtech.2023.09.007 PMC10939978

[35] D. Rommel , M. Mork , S. Vedaraman , C. Bastard , L. P. B. Guerzoni , Y. Kittel , R. Vinokur , N. Born , T. Haraszti , L. D. Laporte , Adv. Sci. 2022, 9, 2103554. 10.1002/advs.202103554 PMC8981485 35032119

[36] D. Rommel , S. Vedaraman , M. Mork , L. D. Laporte , JoVE 2022, 184, 64010. 10.3791/64010 35786610

[37] A. Donev , I. Cisse , D. Sachs , E. A. Variano , F. H. Stillinger , R. Connelly , S. Torquato , P. M. Chaikin , Science 2004, 303, 990. 14963324 10.1126/science.1093010

[38] L. Riley , G. Wei , Y. Bao , P. Cheng , K. L. Wilson , Y. Liu , Y. Gong , T. Segura , Small 2023, 19, 2303466. 10.1002/smll.202303466 PMC10592564 37267936

[39] L. Zhang , G. Feng , Z. Zeravcic , T. Brugarolas , A. J. Liu , D. Lee , ACS Nano 2013, 7, 8043. 23971916 10.1021/nn403214p

[40] A. C. Suturin , A. J. D. Krüger , K. Neidig , N. Klos , N. Dolfen , M. Bund , T. Gronemann , R. Sebers , A. Manukanc , G. Yazdani , Y. Kittel , D. Rommel , T. Haraszti , J. Köhler , L. De Laporte , Adv. Healthcare Mater. 2022, 11, 2200989. 10.1002/adhm.202200989 PMC11469137 36100464

[41] D. E. Stornello , J. Kim , Z. Chen , K. Heaton , T. H. Qazi , ACS Biomater. Sci. Eng. 2025, 11, 1242. 39788546 10.1021/acsbiomaterials.4c02102 PMC11817678

[42] J. O. Freeman , S. Peterson , C. Cao , Y. Wang , S. V. Franklin , E. R. Weeks , Granular Matter 2019, 21, 84.

[43] A. Wouterse , S. R. Williams , A. P. Philipse , J. Phys.: Condens. Matter 2007, 19, 406215. 22049114 10.1088/0953-8984/19/40/406215

[44] V. G. Muir , T. H. Qazi , S. Weintraub , B. O. Torres Maldonado , P. E. Arratia , J. A. Burdick , Small 2022, 18, 2201115. 10.1002/smll.202201115 PMC9463088 35315233

[45] T. Farjami , A. Madadlou , Food Hydrocolloids 2017, 62, 262.

[46] Q. Feng , D. Li , Q. Li , X. Cao , H. Dong , Bioact. Mater. 2022, 9, 105. 34820559 10.1016/j.bioactmat.2021.07.020 PMC8586262

[47] P. Panda , S. Ali , E. Lo , B. G. Chung , T. A. Hatton , A. Khademhosseini , P. S. Doyle , Lab Chip 2008, 8, 1056. 18584079 10.1039/b804234a PMC2790079

[48] H. Zhang , C. Li , Y. Zhang , C. An , H. Li , J. Yu , Y. Zhang , W. He , H. Wang , Front. Sens. 2022, 3, 1037723.

[49] E. L. Pardue , S. Ibrahim , A. Ramamurthi , Organogenesis 2008, 4, 203. 19337400 10.4161/org.4.4.6926 PMC2634325

[50] D. Park , Y. Kim , H. Kim , K. Kim , Y.‐S. Lee , J. Choe , J.‐H. Hahn , H. Lee , J. Jeon , C. Choi , Y.‐M. Kim , D. Jeoung , Mol. Cells 2012, 33, 563. 22610405 10.1007/s10059-012-2294-1 PMC3887750

[51] F. Bonafè , M. Govoni , E. Giordano , C. M. Caldarera , C. Guarnieri , C. Muscari , J. Biomed. Sci. 2014, 21, 100. 25358954 10.1186/s12929-014-0100-4 PMC4226915

[52] W. L. Olbricht , Annu. Rev. Fluid Mech. 1996, 28, 187.

[53] L. Riley , E. C. Lee , P. Cheng , D. A. Alvarez , T. Segura , bioRxiv 2025 666315.

[54] Y. Liu , A. Suarez‐Arnedo , L. Riley , T. Miley , J. Xia , T. Segura , Adv. Healthcare Mater. 2023, 12, 2300823. 10.1002/adhm.202300823 PMC10592513 37165945

[55] H. Malektaj , S. Nour , R. Imani , M. H. Siadati , Int. J. Pharm. 2023, 643, 123233. 37460050 10.1016/j.ijpharm.2023.123233

[56] S. Singh , S. Prakash , S. K. Gupta , Mol. Ther.– Nucleic Acids 2022, 29, 88. 35795481 10.1016/j.omtn.2022.06.007 PMC9249573

[57] T. E. Robinson , E. A. B. Hughes , A. Bose , E. A. Cornish , J. Y. Teo , N. M. Eisenstein , L. M. Grover , S. C. Cox , Adv. Healthcare Mater. 2020, 9, 1901521. 10.1002/adhm.201901521 31977153

[58] M. Nguyen , M. Karkanitsa , K. L. Christman , Nat. Rev. Bioeng. 2024, 2, 810.

[59] D. S. Li , R. Avazmohammadi , C. B. Rodell , E. W. Hsu , J. A. Burdick , J. H. Gorman , R. C. Gorman , M. S. Sacks , Acta Biomater. 2020, 114, 296. 32739434 10.1016/j.actbio.2020.07.046 PMC7484038

[60] J. Fang , J. Koh , Q. Fang , H. Qui , M. M. Archang , M. M. Hasani‐Sadrabadi , H. Miwa , X. Zhong , R. Sievers , D.‐W. Gao , R. Lee , D. D. Carlo , S. Li , Adv. Funct. Mater. 2020, 30, 2004307. 33708028 10.1002/adfm.202004307 PMC7942842

[61] S. Ramadan , N. Paul , H. E. Naguib , Biomed. Mater. 2017, 12, 025013. 28065929 10.1088/1748-605X/aa57a5

[62] A. Jaberi , A. Kedzierski , S. Kheirabadi , Y. Tagay , Z. Ataie , S. Zavari , M. Naghashnejad , O. Waldron , D. Adhikari , G. Lester , C. Gallagher , A. Borhan , D. Ravnic , E. Tabdanov , A. Sheikhi , Adv. Healthcare Mater. 2024, 13, 2402489. 10.1002/adhm.202402489 PMC11828485 39152936

[63] A. Solsona‐Pujol , N. Di Caprio , H. M. Zlotnick , M. D. Davidson , M. B. Riffe , J. A. Burdick , J. Biomed. Mater. Res., Part A 2025, 113, 37889. 10.1002/jbm.a.37889 PMC12254942 40033794

[64] A. Fink , D. B. Brückner , C. Schreiber , P. J. F. Röttgermann , C. P. Broedersz , J. O. Rädler , Biophys. J. 2020, 118, 552. 31864660 10.1016/j.bpj.2019.11.3389 PMC7002917

[65] H. Sunami , Y. Shimizu , H. Kishimoto , Biophys. Physicobiol. 2024, 21, 210004. 10.2142/biophysico.bppb-v21.0004 PMC11128307 38803333

[66] J. L. Ifkovits , E. Tous , M. Minakawa , M. Morita , J. D. Robb , K. J. Koomalsingh , J. H. Gorman , R. C. Gorman , J. A. Burdick , Proc. Natl. Acad. Sci. U. S. A. 2010, 107, 11507. 20534527 10.1073/pnas.1004097107 PMC2895138

[67] L. V. Le , P. Mohindra , Q. Fang , R. E. Sievers , M. A. Mkrtschjan , C. Solis , C. W. Safranek , B. Russell , R. J. Lee , T. A. Desai , Biomaterials 2018, 169, 11. 29631164 10.1016/j.biomaterials.2018.03.042 PMC5931400

[68] B. P. Purcell , J. A. Elser , A. Mu , K. B. Margulies , J. A. Burdick , Biomaterials 2012, 33, 7849. 22835643 10.1016/j.biomaterials.2012.07.005 PMC3449064

[69] S. J. Yoon , Y. H. Fang , C. H. Lim , B. S. Kim , H. S. Son , Y. Park , K. Sun , J. Biomed. Mater. Res., Part B 2009, 91B, 163. 10.1002/jbm.b.31386 19399850

[70] K. L. Aya , R. Stern , Wound Repair Regener. 2014, 22, 579. 10.1111/wrr.12214 25039417

[71] S. Ibrahim , A. Ramamurthi , J. Tissue Eng. Regener. Med. 2008, 2, 22. 10.1002/term.61 18265428

[72] J. Karam , B. J. Singer , H. Miwa , L. H. Chen , K. Maran , M. Hasani , S. Garza , B. Onyekwere , H.‐C. Yeh , S. Li , D. D. Carlo , S. K. Seidlits , Acta Biomater. 2023, 169, 228. 37572983 10.1016/j.actbio.2023.08.001 PMC11729822

[73] J. E. Rayahin , J. S. Buhrman , Y. Zhang , T. J. Koh , R. A. Gemeinhart , ACS Biomater. Sci. Eng. 2015, 1, 481. 26280020 10.1021/acsbiomaterials.5b00181 PMC4533115

[74] P. Mohindra , J. X. Zhong , Q. Fang , D. L. Cuylear , C. Huynh , H. Qiu , D. Gao , B. N. Kharbikar , X. Huang , M. L. Springer , R. J. Lee , T. A. Desai , npj Regener. Med. 2023, 8, 60. 10.1038/s41536-023-00336-w PMC10593781 37872196

[75] W. M. Gramlich , I. L. Kim , J. A. Burdick , Biomaterials 2013, 34, 9803. 24060422 10.1016/j.biomaterials.2013.08.089 PMC3830935

[76] S. Bolte , F. P. Cordelieres , J. Microsc. 2006, 224, 213. 17210054 10.1111/j.1365-2818.2006.01706.x

