# Supplementary material for: Microgel Aspect Ratio Influences Injectable Granular Hydrogel Scaffold Pore Structure and Cellular Invasion for Tissue Repair
Source: Adv Sci (Weinh). 2025 Sep 12;12(43):e11513. doi: 10.1002/advs.202511513 (PMC12631905; doi:10.1002/advs.202511513)
Supplement: Supplementary file 1 — Supporting Information [file ADVS-12-e11513-s001.pdf]

Supplementary Information

Microgel aspect ratio influences injectable granular hydrogel scaffold pore structure and cellular invasion for tissue repair

Gabriel J. Rodriguez-Rivera[1,7], Siddharth Sharma[2], Chima Maduka[1], Sara Boyd[3], Amy Rutledge Perry[1], Nikolas Di Caprio[1,4], Lindsay Riley[5], Connor E. Miksch[5], Daeyeon Lee[2], Tatiana Segura[5], David Issadore[4], Jason A. Burdick[1,4,6]

Affiliations:
[1]BioFrontiers Institute, University of Colorado Boulder, Boulder, CO 80309, USA
[2]Department of Chemical and Biomolecular Engineering, University of Pennsylvania, Philadelphia, PA 19104, USA
[3]Materials Science & Engineering Program, University of Colorado Boulder, Boulder, CO 80309, USA
[4]Department of Bioengineering, University of Pennsylvania, Philadelphia, PA 19104, USA
[5]Department of Biomedical Engineering, Duke University, Durham, NC 27708
[6]Department of Chemical and Biological Engineering, University of Colorado Boulder, Boulder, CO 80309, USA
[7]Present location: Department of Chemical and Biochemical Engineering, Villanova University, Villanova, PA 19085, USA

Supplementary Figures

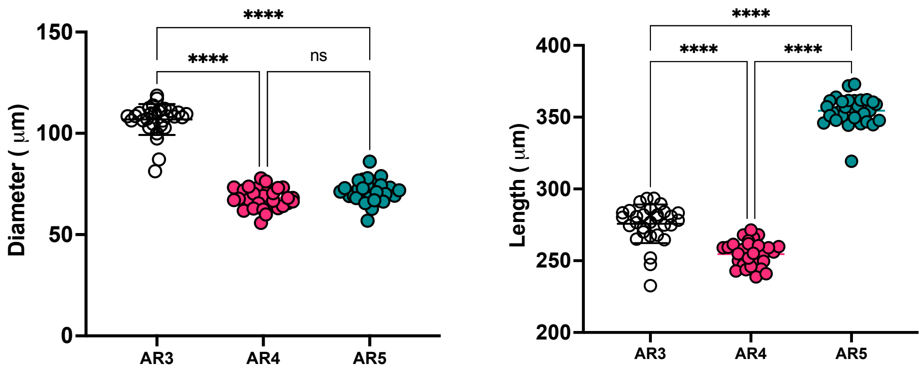

Supplementary Figure 1. Dimensions of rod-like microgels, with approximate aspect ratios of 3 (AR3), 4 (AR4), and 5 (AR5). (n=30); Mean (SD); ns: not significant, $p > 0.05$; ****$p < 0.0001$.

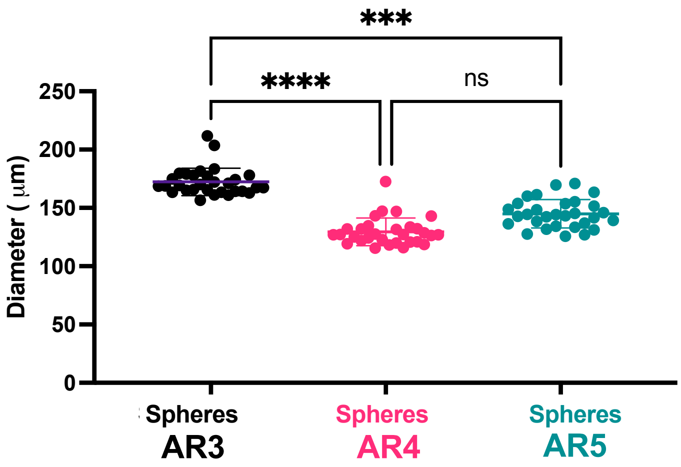

Supplementary Figure 2. Dimensions of spherical microgels, with volumes that match their corresponding rod-like microgels. (n=30); Mean (SD); ns: not significant, $p > 0.05$; ***$p < 0.001$; ****$p < 0.0001$.

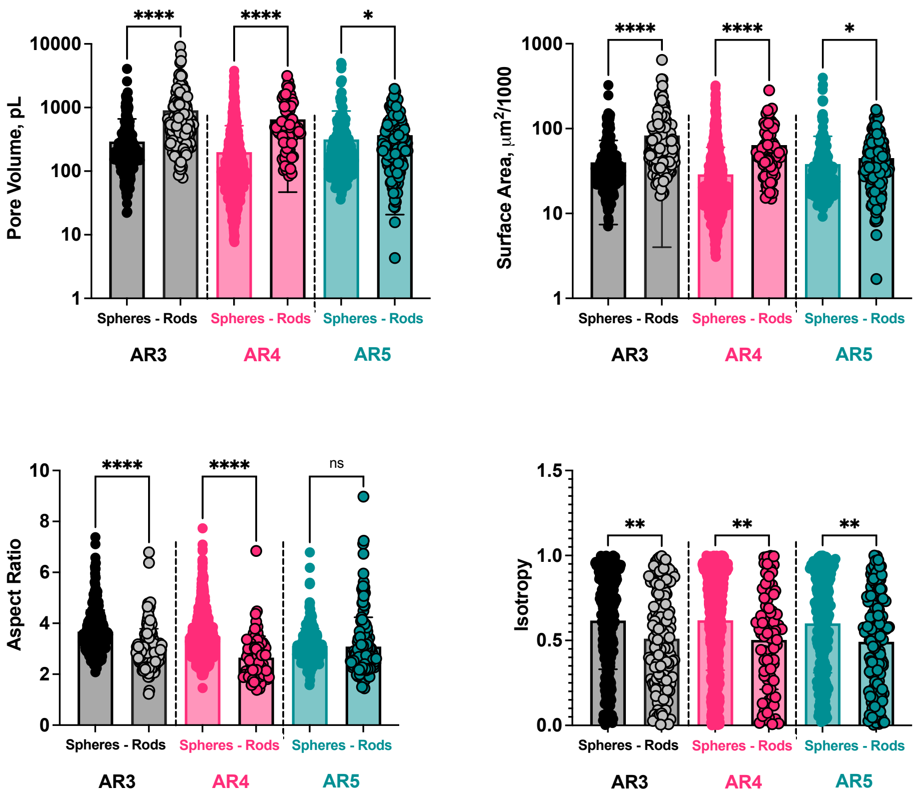

Supplementary Figure 3. Pore descriptors for granular hydrogel scaffolds from rod-like microgels of varied aspect ratios and spherical controls. Descriptors and quantification of individual pores segmented by LOVAMAP, including: A) pore volume, B) surface area, C) aspect ratio, and D) isotropy. The number of internal pores identified by LOVAMAP varies across samples (3 independent scaffolds, 2 locations/scaffold, Spheres AR3: n=335, Rods AR3: n=152, Spheres AR4: n=949, Rods AR4: n=80, Spheres AR5: n=281, Rods AR5: n=166). Mean (SD); ns: not significant, $p > 0.05$; *$p < 0.05$; **$p < 0.01$; ****$p < 0.0001$.

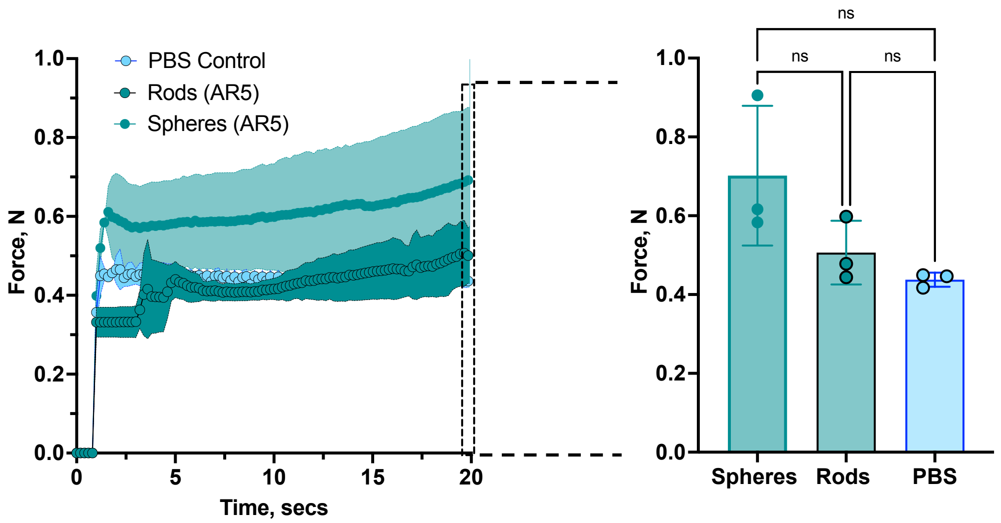

Supplementary Figure 4. Forces recorded during granular hydrogel extrusion. Average force profiles (left) and maximum forces (right) for the extrusion of PBS control or granular hydrogels from rod-like (AR5) or spherical microgels from a 1mL syringe (27G, 1 1/4" needle). (n=3); Mean (SD); ns: not significant, $p > 0.05$.

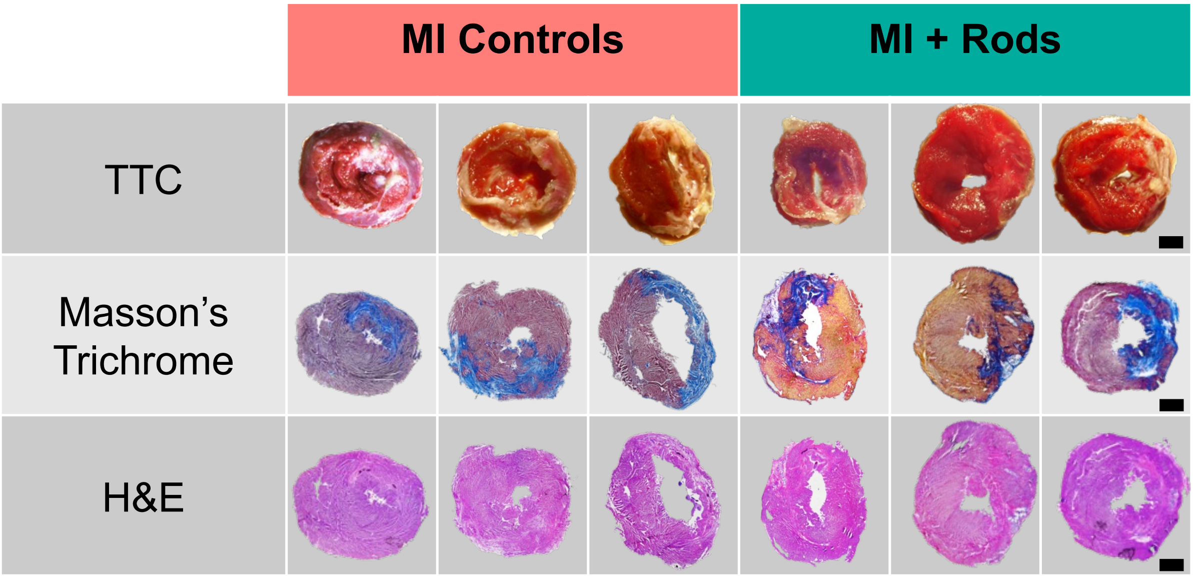

Supplementary Figure 5. Biological replicates of heart tissue after MI without or with treatment. Representative images of tissue sections and staining from three distinct hearts from MI controls or MI treated with granular hydrogels from rod-like microgels, showing the infarct (Triphenyl tetrazolium chloride (TTC) staining) and fibrotic (Masson's trichrome stain (MTS)) areas, as well as histological architecture (Hematoxylin and eosin (H&E) stain). Note that the hydrogel is lost during sectioning for the MTS and H&E stains. Scale bar = 2 mm.

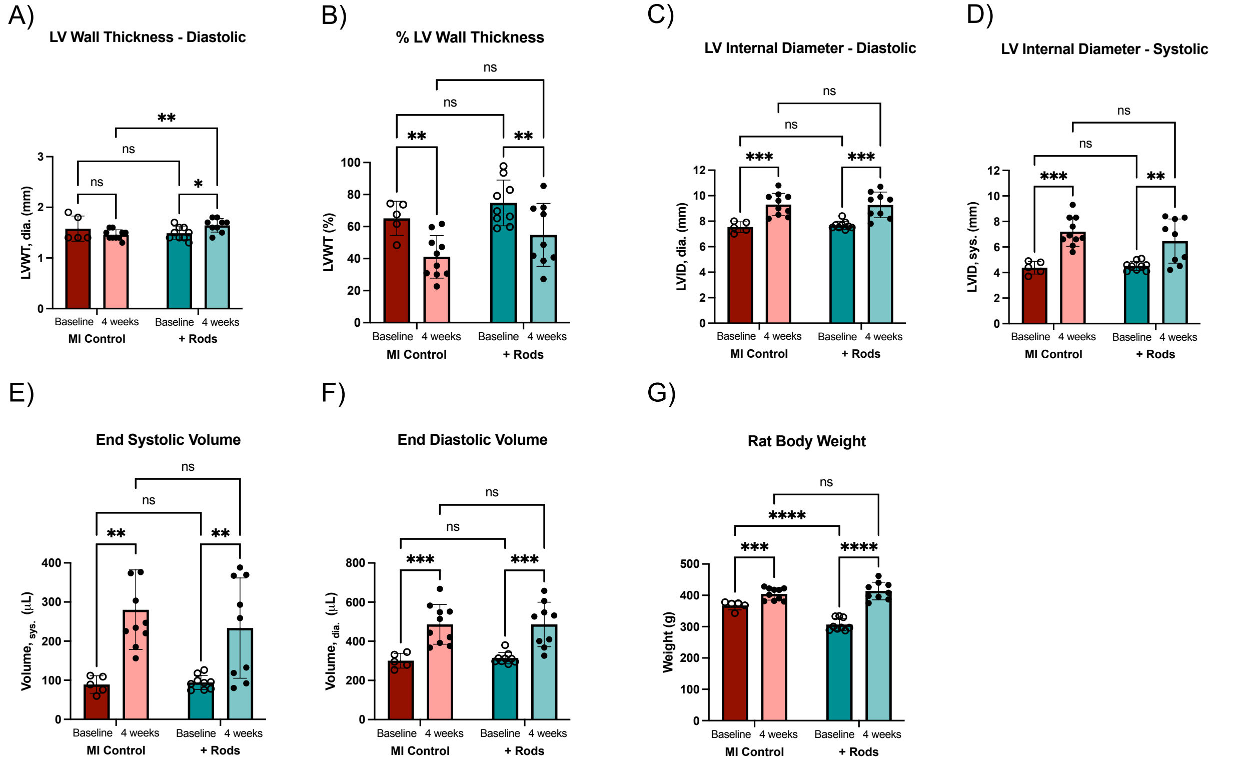

Supplementary Figure 6. Echocardiography data for control MI and MI treatment with granular hydrogels from rod-like microgels. Echocardiography data showing the impact of control and treatment groups on: A) Left Ventricular Wall Thickness – Diastolic (LVWT, dia.), B) % change between diastolic and systolic (LVWT (%), C) Left Ventricular Internal Diameter-Diastolic (LVWT, dia.), D) Left Ventricular Internal Diameter-Diastolic (LVWT, sys.), E) End Systolic Volume (Volume, sys.), F) End Diastolic Volume (Volume, dia.), and G) Rat body weight. The baseline was taken before the MI procedure. Mean (SD); ns: not significant, $p > 0.05$; $^{*}p < 0.05$; $^{**}p < 0.01$; $^{***}p < 0.001$; $^{****}p < 0.0001$.

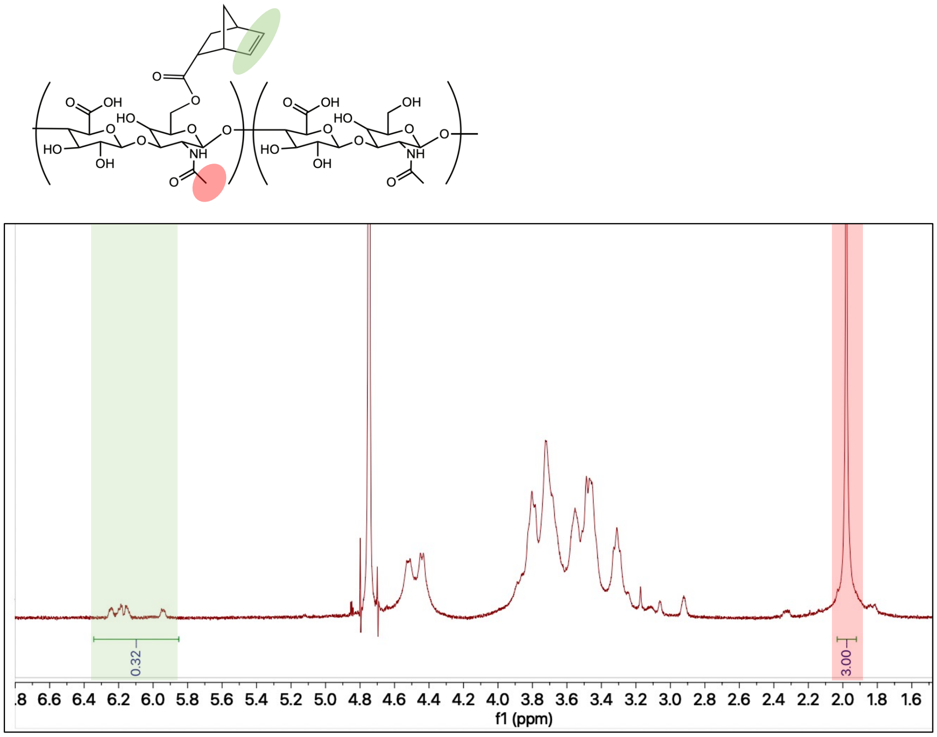

Supplementary Figure 7. ^{1}H NMR Spectra of norbornene-modified hyaluronic acid (NorHA). Norbornene modification (~16%) of HA was determined by integration of the vinyl peaks (2H, shaded green) relative to the methyl group of HA (3H, shaded red).

Supplementary Table

Source of Variation	% of total variation	P value	P value	Significant?
AR	19.21	0.0001	***	Yes
Shape	71.46	<0.0001	****	Yes
Interaction	3.867	0.0403	*	Yes

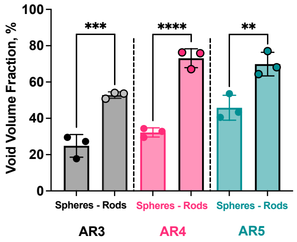

Supplementary Table 1. Two-way ANOVA of the impact of AR and shape on void volume fraction. Summary of p-values and contributions to data variation (also referred to as correlation ratio) for AR factor (3 – 5) and Shape factor (Spheres vs. Rods) and their interaction. *$p < 0.05$; ***$p < 0.001$; ****$p < 0.0001$.
